# Chemerin Impact on Alternative mRNA Transcription in the Porcine Luteal Cells

**DOI:** 10.3390/cells11040715

**Published:** 2022-02-17

**Authors:** Karol G. Makowczenko, Jan P. Jastrzebski, Lukasz Paukszto, Kamil Dobrzyn, Marta Kiezun, Nina Smolinska, Tadeusz Kaminski

**Affiliations:** 1 Department of Animal Anatomy and Physiology, Faculty of Biology and Biotechnology, University of Warmia and Mazury in Olsztyn, Oczapowskiego 1A, 10-719 Olsztyn, Poland; karol.makowczenko@uwm.edu.pl (K.G.M.); marta.kiezun@uwm.edu.pl (M.K.); nina.smolinska@uwm.edu.pl (N.S.); 2 Department of Plant Physiology, Genetics and Biotechnology, Faculty of Biology and Biotechnology, University of Warmia and Mazury in Olsztyn, Oczapowskiego 1A, 10-719 Olsztyn, Poland; bioinformatyka@gmail.com; 3 Department of Botany and Nature Protection, Faculty of Biology and Biotechnology, University of Warmia and Mazury in Olsztyn, Plac Lodzki 1, 10-719 Olsztyn, Poland; lukasz.paukszto@uwm.edu.pl; 4 Department of Zoology, Faculty of Biology and Biotechnology, University of Warmia and Mazury in Olsztyn, Oczapowskiego 5, 10-719 Olsztyn, Poland; kamil.dobrzyn@uwm.edu.pl

**Keywords:** corpus luteum, pig, lncRNA, alternative splicing, allele-specific, hormone, luteal phase, reproduction, transcription, expression

## Abstract

Chemerin participates in the regulation of processes related to physiological and disorder mechanisms in mammals, including metabolism, obesity, inflammation, and reproduction. In this study, we have investigated chemerin influence on alternative mRNA transcription within the porcine luteal cell transcriptome, such as differential expression of long non-coding RNAs (DELs) and their interactions with differentially expressed genes (DEGs), differences in alternative splicing of transcripts (DASs), and allele-specific expression (ASEs) related to the single nucleotide variants (SNVs) frequency. Luteal cells were collected from gilts during the mid-luteal phase of the oestrous cycle. After in vitro culture of cells un-/treated with chemerin, the total RNA was isolated and sequenced using the high-throughput method. The in silico analyses revealed 24 DELs cis interacting with 6 DEGs and trans-correlated with 300 DEGs, 137 DASs events, and 18 ASEs. The results enabled us to analyse metabolic and signalling pathways in detail, providing new insights into the effects of chemerin on the corpus luteum functions related to inflammatory response, leukocyte infiltration, the occurrence of luteotropic and luteolytic signals (leading to apoptosis and/or necroptosis). Validation of the results using qPCR confirmed the predicted expression changes. Chemerin at physiological concentrations significantly modifies the transcription processes in the porcine luteal cells.

## 1. Introduction

Chemerin (CHEM) is a hormone discovered in 1997 during studies analysing the pathogenesis of psoriasis as a product of the *RARRES2* gene (originally known as *TIG2*; short names of genes and macromolecules are explained in the Abbreviations part), whose expression was increased in response to retinoid substances [1]. In 2007, due to its metabolic functions and high secretion by the adipose tissue, CHEM was classified as an adipokine [2,3]. More recently, it was also categorized as a cytokine due to its involvement in immune processes, such as tissue-dependent pro- or anti-inflammatory activity and chemotactic activity on leukocytes, particularly macrophages, dendritic and NK cells [3,4]. Three membrane receptors capable of binding CHEM molecules have been identified in mammals—CMKLR1 (also referred to as ChemR23), GPR1 and CCRL2 [5]. The first two receptors bind the N-terminus of CHEM with similar high affinity and, through binding to G–proteins, cause signal transduction dependent on the PI3K/Akt and MAPK–ERK1/2 (CMKLR1) and AMPK (CMKLR1 and GPR1) pathways [3,5,6]. In contrast, CCRL2, called “atypical receptor”, binds the C-terminus of CHEM with much lower affinity, does not induce signal transduction, and participates in local CHEM aggregation and exposition to the two remained receptors—CMKLR1 and GPR1 [7,8].

Around 2012, attention was paid to the influence of CHEM on the reproductive system in mammals, focusing primarily on its effects on the granulosa cells (GCs) steroidogenic activity in humans and rats [9,10,11], and role in the early stages of pregnancy [12,13]. Since 2019, we have reported the presence of CHEM and its receptors in all branches of the hypothalamic–pituitary–gonadal (HPG) regulatory axis and in the reproductive system of mature gilts, both during different phases of the oestrous cycle and early pregnancy. The hormone was found in the selected areas of hypothalamus [14], anterior and posterior pituitary [15,16], ovarian structures, such as follicles and corpora lutea (CLs) [17], and uterus [18]. We have recently described the effects of CHEM on the transcriptomic profile of porcine luteal cells (LCs) and direct implications for the CLs secretory functions. We have shown CHEM effects on steroidogenesis and prostaglandin synthesis essential for ovarian function, on activation of NF-κB and Jak/STAT signalling pathways, pro- and anti-apoptotic activities, as well as angiogenesis [19,20,21].

It is now often reiterated that gene expression affects at least 40% of the variation in proteins produced in mammalian cells [22,23]. However, considering experimental errors, changes at the mRNA level explain up to 68–84% of the variance at the protein level in animal and human cells [24,25,26]. We would like to emphasize that, apart from the study of gene expression differences alone, it is equally important to study the phenomena accompanying transcriptional or posttranscriptional modifications. This gives a better overview of the complex processes taking place in the studied cells and, in a broader perspective, a better description of molecular aspects of physiological mechanisms occurring in tissues and organs.

Long non-coding RNAs (lncRNAs) are the largest transcripts group in the mammalian transcriptomes [27]. They are defined as RNA molecules longer than 200 nt, without noticeable protein-coding potential and are composed of at least two exons. They are progressively being considered as important (positive or negative) regulators of gene expression by controlling transcription, modulating mRNA stability and the course of translation and post-translational modifications [28]. In general, transcription control by lncRNAs can be cis interacting, when regulation occurs within a single chromosome, or trans-interacting, when the target gene is located on another chromosome or far apart on the same chromosome [29]. Furthermore, lncRNAs can affect protein expression directly associated with the target gene, often by binding a complementary sequence, but also in indirect ways, i.e., interacting with the second chromosome of a pair or affecting the expression of RNA polymerases [27]. Interestingly, lncRNAs can modulate the activity of proteins by binding to them [30]. Long non-coding RNAs often exhibit tissue- or cell-specific expression [31]. There are increasing reports that lncRNAs are involved in the regulation of reproductive functions in mammals [32,33], including the proper functioning of CLs [34]. In the course of our research objective, we conducted identification and selection of differentially expressed lncRNAs (DELs) under the effects of CHEM and performed cis-interaction analysis with differentially expressed genes (DEGs), on the basis of colocalization in the genome, and trans-interaction considering expression correlation and similarity of spatial structures between lncRNAs and mRNAs/proteins.

Alternative splicing is a mechanism for producing multiple transcript variants based on a single gene template [35,36]. This phenomenon exploits the alternative splicing sites of transcripts during intron deletion or retention within pre-mRNAs. Alternative splicing by regulating protein folding contributes to increased proteome diversity, and ultimately cellular and functional complexity, without increasing the eukaryotic organisms’ genome size [37]. It is worth noting that the result of the described process may be polymorphism of proteins encoded by a single gene, which contain different domains and, consequently, may exert different biological functions [38]. Alternatively spliced variants may contain premature stop codons. Protein-coding transcripts carrying a nonsense codon upstream of the terminal exon junction may be targeted for degradation. This mechanism serves to modulate the amount of protein products of a gene without altering its expression in the mammalian cells [39]. Additionally, certain clipped proteins may directly antagonize the full-length proteins activity by retain domains capable of interacting with protein complexes or substrates [40]. Significantly, expression of alternatively splicing transcript variants is often tissue specific [41]. Alternative splicing within genes which products are involved in luteolysis, maternal recognition of pregnancy, and vascular growth and regression is observed in CLs [42,43]. In this publication, we performed a detailed identification of differential alternative splicing events (DASs) appearing in CHEM-treated and native LCs.

Most genes are expressed equally from both alleles; however, some genes are differentially expressed in an allele-dependent manner. Under the influence of regulatory factors such as DNA methylation, histone modifications, and non-coding RNA activity, the production of a protein encoded by one of the alleles of the same gene can be promoted in heterozygotes [44]. This phenomenon is an important genetic factor leading to phenotypic variation induced by, among others, variable environmental conditions [45]. The intensity of angiogenesis in humans has been linked to the occurrence of specific single nucleotide polymorphisms directly related to plasma CHEM levels [46]. This leads to the conclusion that the hormone analysed in the present study is capable of promoting the expression of one of the allelic variants in the affected cells. Allele-specific expression variants (ASEs) were detected out of single nucleotide variants (SNVs) showing imbalance ratios within DEGs.

Despite extensive research analysing the general mechanisms of CHEM action, to date, the details of its influence at the molecular level on specific cells resulting in the modulation of transcription and translation processes remains poorly described. This manuscript is the extension of our previously published analyses of transcriptional profiles on the same research model [19]. Moreover, we have analysed the results obtained by our team using other laboratory techniques on identical research models [17,20,21]. The purpose of this study was to investigate in detail the molecular mechanisms by which CHEM can influence and regulate the transcriptional processes in porcine LCs during the mid-luteal phase of the oestrous cycle. In pursuit of this objective, we focused on alternative mRNA transcription mechanisms, such as DELs interactions with DEGs, as well as DASs within genes, and ASEs within DEGs, which were analysed using high-throughput RNA sequencing data.

## 2. Materials and Methods

### 2.1. Collection of Samples, In Vitro Cell Culture and Chemerin Administration

The experiment was performed on five mature Large White × Polish Landrace female pigs, aged 7–8 months, with a body weight of 120–130 kg, obtained from a private breeding farm. The females, during the mid-luteal phase of the oestrous cycle (days 10–12 of the cycle), were used for the research. Gilts were daily observed for oestrus behaviour in the presence of a boar. The day when the symptoms of the second oestrus appeared was designated as day 0 of the oestrous cycle. Additionally, ovarian morphology was verified to confirm the phase of the cycle [47]. Immediately after humane slaughter, ovaries were collected and transported to the laboratory in ice-cold PBS supplemented with 100 IU/mL penicillin and 100 µg/mL streptomycin.

Luteal cells were isolated from the slaughter material according to the procedure described by Kaminski et al. [48]. Briefly, CLs were dissected from the ovaries, divided into smaller fragments, and dispersed in Ham’s F-12 nutrient mixture (Sigma-Aldrich, St. Louis, MO, USA) containing 1% of bovine serum albumin fraction V (BSA; Sigma-Aldrich, St. Louis, MO, USA) and a mix of antibiotics (Sigma-Aldrich, St. Louis, MO, USA). Swine CLs were enzymatically decomposed 4–6 times using 0.125% trypsin (Sigma-Aldrich, St. Louis, MO, USA) solution at 38 °C, centrifuged for 10 min at 300× *g* at 21 °C, and washed three times. Luteal cells were filtered from trypsinized tissue residues with a 75 µm mesh nylon filter and resuspended in fresh Ham’s F-12 mixture. The number of cells was estimated with a haemocytometer and their viability of ≈90% was determined with 0.4% trypan blue dye (Sigma-Aldrich, St. Louis, MO, USA) exclusion.

**Figure 1:**
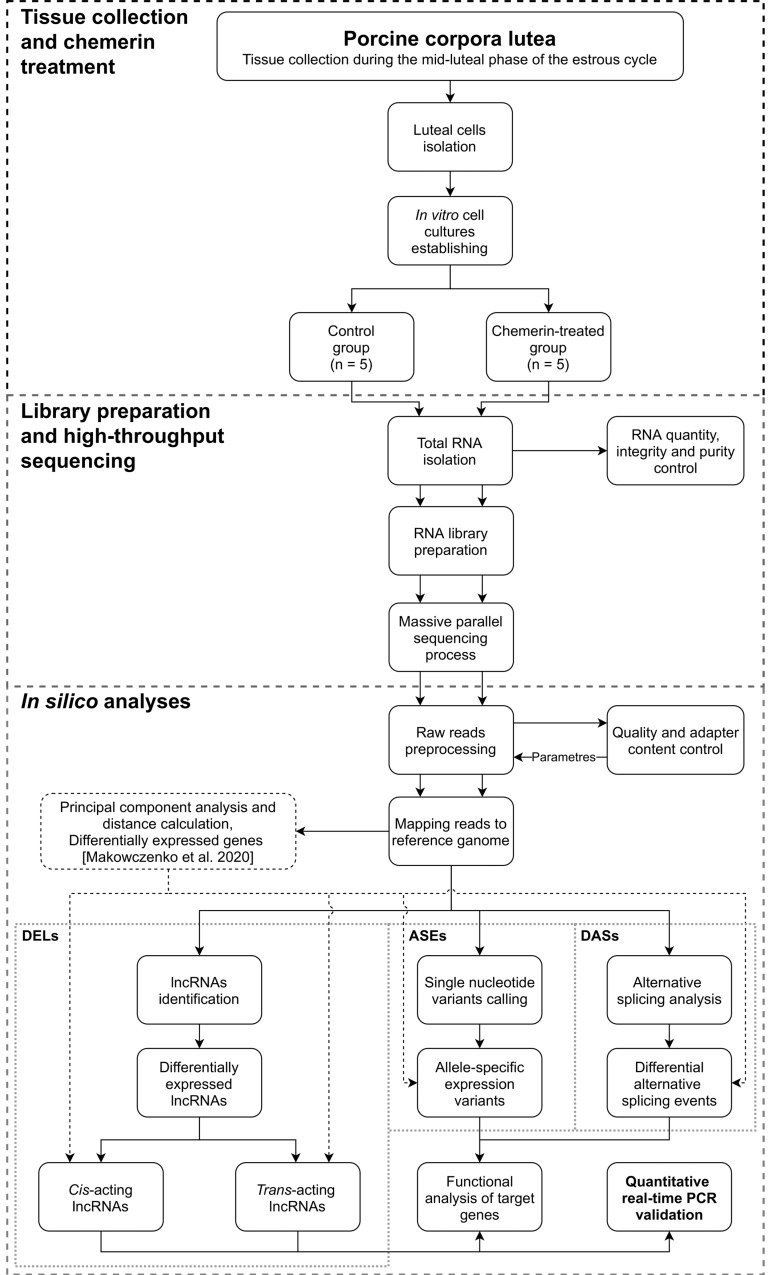
A stage-by-stage pipeline of the research performed within the scope of this manuscript, including the use of the results of differential gene expression analysis performed on the same research model (doi:10.3390/genes11060651). Abbreviations: lncRNAs—long noncoding RNAs, DELs—differentially expressed lncRNAs, ASEs—allele-specific expression variants, DASs—differential alternative splicing events.

Luteal cells were resuspended at a concentration of 2 × 10^6^/2 mL in Ham’s F-12 medium supplemented with 20% foetal bovine serum (Sigma-Aldrich, St. Louis, MO, USA), 1% BSA, and antibiotics. The cells were pre-incubated in a humidified incubator with 95% air and 5% CO_2_ atmosphere for 48 h. The serum-containing culture medium was rejected, and LCs were flushed with serum-free Ham’s F-12 nutrient mixture. Porcine LCs were cultured for 24 h in F-12 mixture with 1% BSA, antibiotics, and with or without the recombinant human CHEM (cat. no. 268-10032; RayBiotech, Peachtree Corners, GA, USA) at the concentration of 200 ng/mL for the experimental and control group (*n* = 5), respectively. The human recombinant CHEM was applied due to the unavailability of porcine CHEM in the onset of laboratory phase of the experiment. In accordance to Du and Leung’s study [49], the porcine and human CHEM amino acid homologs show 84% identity. Furthermore, Luangsay et al. [50] have shown that the C-terminus fragment of the mature protein (YFPGQFAFS) is highly conserved in all mammalian species and is highly important for CHEM biological activity. Rytelewska et al. [21] have shown that the identity of the mentioned short amino acid fragment in mammals equals 89% and suggested that CHEM interaction with the cognate receptors is well conserved across mammalian species. The concentration of CHEM was selected on the basis of its blood plasma level in women [46] and gilts [14]. The whole process of performed analysis is shown in Figure 1.

### 2.2. RNA Isolation, Library Preparation and High-Throughput Sequencing Procedure

From in vitro cultures of LCs treated and untreated with CHEM, total RNA was extracted with the use of RNeasy Mini Kit (Qiagen, Germantown, MD, USA) with DNase included in RNase-free DNase Set (Qiagen, Germantown, MD, USA), in accordance with the manufacturer’s procedure. The purity (A_260_/A_280_) and quantity (wavelength 260 nm, A_260_) of the obtained RNA was estimated spectrophotometrically with an Infinite M200 Pro (Tecan, Männedorf, Switzerland). RNA integrity was tested using the Bioanalyzer 2100 (Agilent Technology, St. Clara, CA, USA). RNA integrity number in a range between 8 and 10 qualified isolated RNA for the transcriptome high-throughput sequencing (RNA-Seq) analysis and the quantitative real-time polymerase chain reaction (qPCR) validations. RNAs extracted from all samples were stored at −80 °C. The strand-specific sequencing libraries were prepared separately from each sample RNA using the TruSeq Stranded mRNA Library Prep Kit (Illumina, San Diego, CA, USA), according to Shen et al. [51] recommendations. The RNA-Seq was performed on the NovaSeq 6000 instrument (Illumina, San Diego, CA, USA). The assumed minimum depth of sequencing was 100 million reads per sample.

### 2.3. In Silico Analyses

The mRNA transcription mechanisms, such as production of lncRNA by cells, alternative splicing of mRNA molecules, and preference for production of mRNA encoded by one of the alleles available in the genetic material, were analysed in order to explore the regulatory mechanisms induced by CHEM in the porcine LCs. The bioinformatic analyses were performed according to Paukszto et al. [52,53], with modifications.

#### 2.3.1. Raw Reads Pre-Processing and Mapping to a Reference Genome

Quality of generated 2 × 150 nt raw paired end reads during sequencing were controlled using FastQC software v. 0.11.8 [54]. Adapters and low-quality regions of raw reads were clipped with the use of Trimmomatic tool v. 0.38 [55] (*Q*_Phred_ score at 5′ and/or 3′ ends < 20; average *Q*_Phred_ score < 30). Reads were trimmed to equal length of 90 nt. Processed reads were rechecked for adapter content and quality with FastQC. The remaining sequences were mapped to the *Sus scrofa* reference genome with Ensembl annotations v. 11.1.99 [56] with the use of STAR mapper v. 2.7.3a [57] with the operating parameters recommended by Jakobi [58]. StringTie software v. 1.3.5 [59] with enabled “fr-firststrand” parameter, was applied to annotate and estimate the expression of genes and transcripts. Counts per gene and per transcript were computed using the *prepDE* Python script provided by the StringTie’s authors [60]. The DEGs analysis process was conducted using the Ballgown tool v. 2.18.0 [61], following the procedure described by Makowczenko et al. [19], with the operating parameters: *q*-value < 0.05 and |log2FC| ≥ 0.5.

#### 2.3.2. Long Non-Coding RNA Analysis

Identification of lncRNAs in the porcine LCs was performed using multistage workflow. Firstly, low-expressed and protein-coding transcripts were removed from the processed dataset. Secondly, one-exonic and short (length < 200 nt) transcripts were excluded. The coding potential of the remaining transcripts was estimated using four tools: the coding potential assessment tool (CPAT) v. 1.2.4 [62], the coding potential calculator 2 (CPC2) v. 2.0.1 [63], the flexible extraction of lncRNAs (Feelnc) [64], and the predictor of lncRNAs and mRNAs based on an improved *k*-mer scheme (PLEK) v. 0.2 [65]. Simultaneously, transcripts’ sequences were aligned into the protein families (Pfam) database v. 33.1 [66] using “hidden Markov model”-based HMMER software v. 3.3.2 [67]. The transcripts found to be devoid of coding potential (CPAT score < 0.364; CPC2 score < 0; Feelnc coding potential < 0.558; PLEK label = “noncoding”) by three of the mentioned tools and with low similarity to Pfam records (*e*-value > 10^−3^) were used in subsequent stages of analysis. The differences in detected lncRNAs expression between CHEM-treated and control samples were computed with the Ballgown tool v. 2.18.0 [61] using binominal test, with a cut-off *q*-value < 0.05 and |log_2_(fold change)| > 0.5. Moreover, the sequences of the obtained DELs were aligned to small RNA model records of the RNA families (Rfam) database v 14.4 [68] with the use of Infernal cmscan v 1.1.3 tool [69].

A further analysis was conducted in R environment v. 4.0.3 [70], with an integrated high-throughput genomic data analysis toolkit—Bioconductor v. 3.12 [71]. It was performed to discover the relationships between the obtained DELs, and mRNAs encoded by DEGs. The relatively close proximity of DELs–DEGs located on the same chromosome were described as cis-interactions. The pairs located in different parts of the genome were also characterized according to transcriptional profiles (trans-action). Analysis of cis-actions was performed within two searching regions on the porcine genome: overlapping, where lncRNA and mRNA-coding genes were located in direct vicinity, and distant, where lncRNA genes were located upstream or downstream of the mRNA-coding genes at a distance of up to 10,000 bp. Trans-actions between DELs and DEGs transcriptional profiles were examined using Pearson’s correlation coefficient, with a cut-off |*r*| > 0.9 and *p*-value < 0.05. In addition, the binding strengths of lncRNAs to potential mRNA targets were tested using LncTar v. 1.0 software [72] with the normalized free energy (ndG) calculation. Moreover, the probability of hydrogen-bonding propensities, and van der Waal’s interaction between the secondary structures of the detected lncRNAs and potential protein targets were calculated using lncPro v. 1.0 tool [73]. In subsequent analyses, only those trans-actions for which LncTar indicated an ndG < −0.1 or lncPro showed a probability > 0.9 were included, providing more detailed information on the DEL–DEG trans-actions detected in the previous steps of the analysis. The identified interactions between DELs and DEGs were visualized using Cytoscape software v. 3.8.3 [74].

#### 2.3.3. Differential Alternative Splicing Events Analysis

Alternative splicing events were predicted with the super-fast pipeline for alternative splicing analysis 2 (SUPPA2) tool v. 2.3 [75]. Briefly, equal length processed reads uniquely mapped against the reference genome were retrieved, and, subsequently, were re-mapped against *S. scrofa* reference transcriptome using Salmon mapper v. 1.1.0 [76]. Differential alternative splicing events between experimental and control groups were statistically tested and the percentage of splicing inclusions (PSI) for all splicing events was calculated. Detected DASs were considered as statistically significant with *p*-value < 0.05, and |ΔPSI| > 0.1. All discovered DASs were classified into seven categories by SUPPA2: alternative 5′ splice site (A5), alternative 3′ splice site (A3), mutually exclusive exons (MX), retained intron (RI), skipping exon (SE), alternative first exon (AF) and alternative last exon (AL). Significant DASs were visualized using the *ggsashimi* Python tool v. 0.5.0 [77].

#### 2.3.4. Single Nucleotide Variants Identification and Allele-Specific Expression Variants Analysis

The single nucleotide variants in transcripts were discovered by aligning RNA-Seq reads to the genome reference sequence. Calling of SNVs with multi-sample data was conducted using the pipeline consisting of Picard software v. 2.6.0 [78], the replicate multivariate analysis of transcript splicing and discovery of differential variants in RNA (rMATS-DVR) tool v. 1.0.0 [79], and the gold standard genome analysis toolkit (GATK) v. 3.6.0 [80]. Known genomic positions of porcine SNVs were obtained from Ensembl database v. 11.1.99 [56]. All previously created BAM files were recalibrated with the Picard tool. The individual rMATS-DVR modules firstly identified the potential occurrence of SNVs in all sample replicates, and then detected the differences in allele frequencies between CHEM-treated and control sample groups. Low-quality and disrupted SNVs were filtered out on the basis of GATK standard parameters: total depth of base coverage (>10), root mean square mapping quality (>40), quality by depth (>2), mapping quality rank sum (>−12.5), rank sum test for relative positioning of reference versus alternative alleles within reads (>−8). Single nucleotide variants with alternative allele fraction (AAF) > 0 within at least half of the RNA-Seq samples were selected for further analyses. Allelic expression bias of discovered SNVs between experimental and control groups were considered as statistically significant with |ΔAAF| > 0.1 and false discovery rate (FDR) < 0.05. An allelic imbalance ratio of ASEs candidates was confirmed using the ꭕ^2^ goodness-of-fit test (*p*-value < 0.05). The exact location of identified ASEs within genes’ regions and the effect of single nucleotide mutations on the transcription process of specific proteins were determined using the Ensembl variant effect predictor (VEP) web tool [81].

#### 2.3.5. Functional Annotation of Target Genes

To analyse the functions and involvement of potential target DEGs of the identified DELs, DASs and ASEs in biological processes, the Ko-based annotation system (KOBAS) web tool v. 3.0 [82] searching gene ontology (GO) [83,84], the Reactome Knowledgebase [85], and the Kyoto encyclopaedia of genes and genomes (KEGG) [86] databases was implemented. The gene enrichment was performed for every analysed phenomenon individually, with the use of Fisher’s exact test with Benjamini and Hochberg correction (FDR < 0.05). Furthermore, KEGG analysis of target genes were clustered to generate new molecular network interaction maps using a Pathview v. 1.30.1 R package [87]. The statistical significance values were recalculated for the regrouped data (FDR < 0.05).

### 2.4. Quantitative Real-Time PCR Validation

To validate the results discovered during DELs analysis, the qPCR method was applied. The 500 ng of each RNA sample used in the RNA-Seq were rewritten to cDNA, yielding a total volume of 10 µL of each mixture, with the use of Omniscript RT Kit (Qiagen, Germantown, MD, USA), and 0.5 µg of oligo(dT) (Roche, Penzberg, Germany). The reaction of reverse transcription was conducted at 37 °C for 1 h and was terminated by incubation at 93 °C for 5 min.

The quantitative real-time PCR was performed using an AriaMx Real-Time PCR System (Agilent Technology, St. Clara, CA, USA). The constitutively expressed *ACTB* and *GAPDH* as reference genes were used. Primer sequences for target transcripts (*CL.9638.3*, *CL.12742.3*, *ENSSSCT00000075362* and *ENSSSCT00000078829*) were developed using Primer Express software 3 (Applied Biosystems, Waltham, MA, USA). The primer sequences of reference and target genes are listed in Table 1. Rection mixtures with a final volume of 20 µL consisted of 30 ng prepared cDNA, 200 nM of the forward and reverse primers, 12.5 µL of the Power SYBR Green PCR Master Mix (Applied Biosystems, Waltham, MA, USA) and RNase-free water. Quantitative real-time PCR was performed according to the procedure: preliminary cDNA denaturation and enzymes activation at 95 °C for 3 min, 40 cycles of denaturation at 95 °C for 30 s, annealing at 66 °C for 1 min, and elongation at 72 °C for 45 s. For *ACTB* primers the annealing temperature was lowered to 61 °C, and for *ENSSSCT00000075362* primers preliminary denaturation was extended to 10 min and the annealing temperature was lowered to 60 °C. Negative controls were prepared by replacing the cDNA with water. The reactions were performed in technical duplicate for each sample. The amplification specificity was examined at the end of qPCR by melting-curve analysis.

The relative expression levels of validated transcripts were calculated using the comparative cycle threshold (ΔΔCT) method and normalized using the geometrical means of the reference genes expression [88]. The results of qPCR were statistically processed using Student’s *t*-test with a significant *p*-value < 0.05 in the R environment v. 4.0.3 [70] and were presented as mean values ± standard error of the mean (SEM).

**Table 1:** Primers used for the qPCR validation of RNA-Seq results.

Gene symbol	Primers Sequences	Product Length	Reference
CL.9638.3	F: GGGGCCCTGTAAGGAAACTC	141 bp	[The present study]
	R: TACTTGGCACCAAGCAAGCA		
CL.12742.3	F: AGCGGGCGCAGATTCAT	241 bp	[The present study]
	R: AGCAGAGGGTCATTTCTGGC		
ENSSSCT00000075362	F: GGGTGTTTCCATGCTCAAGA	275 bp	[The present study]
	R: CACAGCCAAGACAGCGAATA		
ENSSSCT00000078829	F: GTGCTTGGAGGGACATGACA	186 bp	[The present study]
	R: TGTCGTTTGAGGGTTCTGGG		
ACTB	F: ACATCAAGGAGAAGCTCTGCTACG	366 bp	[89]
	R: GAGGGGCGATGATCTTGATCTTCA		
GAPDH	F: CCTTCATTGACCTCCACTACATGG	183 bp	[90]
	R: CCACAACATACGTAGCACCAGCATC		

Abbreviations: F—forward; R—reverse.

## 3. Results

### 3.1. Overall Statistics of RNA-Seq Data Mapping

The total number of raw reads obtained across all samples by the deep sequencing of transcriptome was 1,154,494,264, and the total number of reads obtained after the pre-processing step was 1,024,385,352. The total number of processed reads mapped to the reference porcine genome (*S. scrofa* v. 11.1.99) was 1,021,940,610 (Table 2). Of them, 96.25% were mapped uniquely and 3.18% were mapped to multiple loci. Among all RNA-Seq libraries used in this study, the average number of reads’ bases mapped to the coding DNA sequences (CDS) were 59.52%, untranslated regions (UTR) were 23.32%, intronic regions were 3.35%, and intergenic locations were 13.81%. The detailed statistics of the sequencing and pre-processing data, principal component analysis and sample-to-sample distance matrix were summarized by Makowczenko et al. [19].

### 3.2. Long Non-Coding RNA Identification and Cis-/Trans-Acting on Protein-Coding Genes

**Figure 2:**
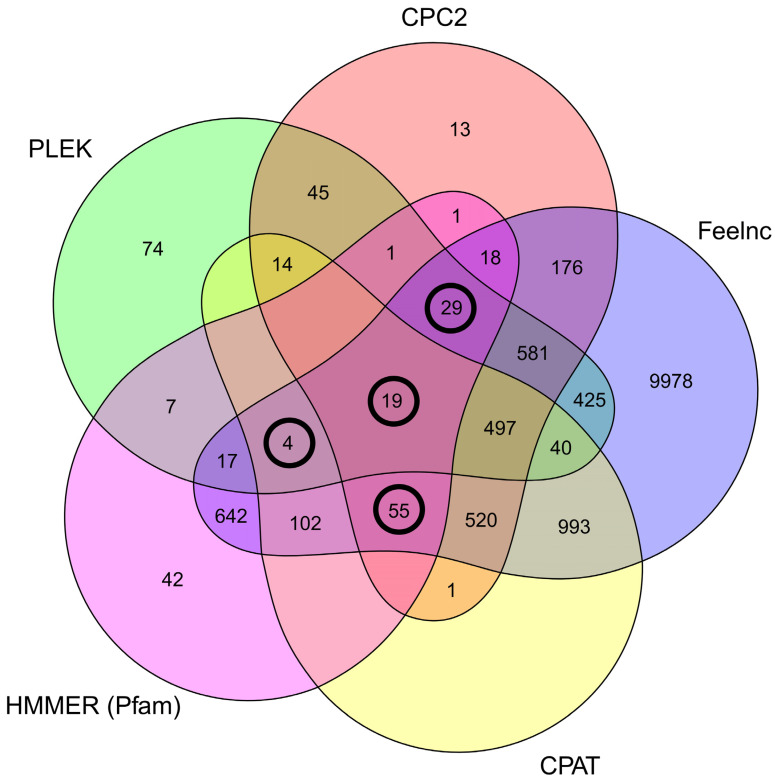
Venn diagram visualizing the relationship between the number of transcripts classified as ‘non-coding’ by each program: CPAT, CPC2, Feelnc, PLEK and HMMER. Values in circles delimited the transcripts used during the subsequent steps of lncRNAs processing. Details are described in the text.

**Figure 3:**
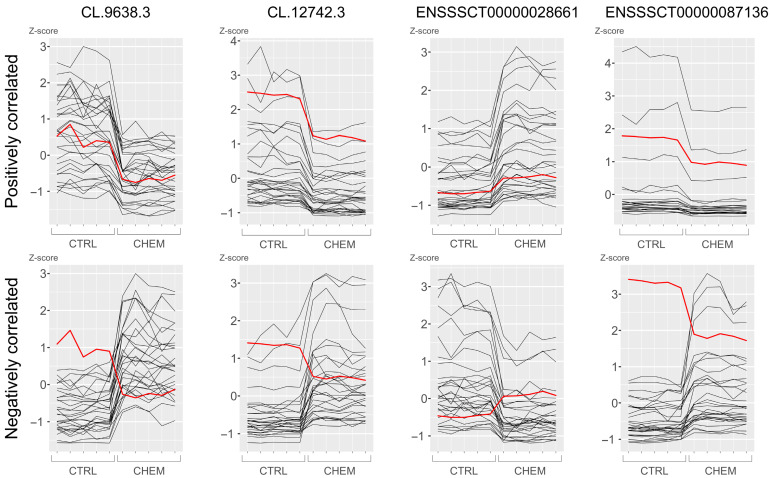
Positive and negative correlations of selected differentially expressed lncRNAs—*CL.9638.3*, *CL.12742.3*, *ENSSSCT00000028661* and *ENSSSCT00000087136* with differentially expressed genes. Red lines indicate lncRNA expression profiles, black lines symbolize gene profiles. To increase readability, samples were grouped and arranged in order 1–5 (from left). Abbreviations: CHEM—chemerin-treated group, CTRL—control group.

The first steps of filtration involved removing from the dataset sequences designated as “protein-coding biotype” in the Ensembl database, as well as single-exon RNAs and RNAs shorter than 200 nt. This process resulted in the detection of 25,252 potential lncRNA candidates in the porcine LCs. Of the surviving transcripts, 9340 were labelled as “lncRNA biotype” in the Ensembl database. The remaining unknown sequences in the data scope were scanned using four tools—CPAT, CPC2, Feelnc and PLEK, and in consequence reducing the number of novel lncRNAs to 1759. Of these, 107 survived alignments to the Pfam database using HMMER tool (Figure 2). Analysis of genes expression differences between CHEM-treated and control samples revealed that 24 of the detected lncRNAs (encoded by 12 genes) undergo expression modifications in LCs under the influence of the CHEM, including 20 previously known and 4 unidentified lncRNAs (Figure 3). Among the identified DELs, 16 were down-regulated while 8 were up-regulated. None of the detected transcripts were matched by Infernal cmscan to small RNA models in the Rfam database. The detected DELs are summarized in Table 3.

**Figure 4:**
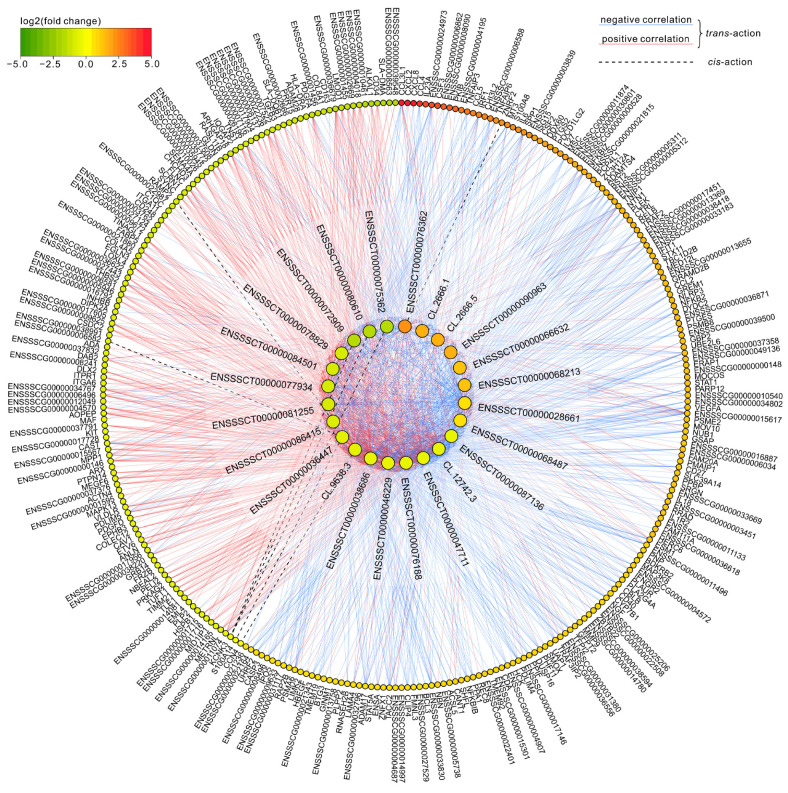
Visualization of interactions occurring between differentially expressed long non-coding RNAs (DELs) and differentially expressed protein-coding genes (DEGs) with expression modified by chemerin in the porcine luteal cells during days 10–12 of the oestrous cycle. Smaller dots in the outer circle symbolize DEGs, larger dots forming the inner circle indicate DELs. Both DEGs and DELs were arranged according to increasing −log2(fold change). Data on DEGs are from another study conducted on the same research model (doi:10.3390/genes11060651).

Cis-interaction analysis revealed 11 cases of DELs affecting 6 DEGs based on their colocalization in the porcine genome. Of these, DELs overlapped target gene loci in 3 interactions, 5 interactions occurred in DELs located within 10,000 bp downstream from DEGs, and 3 interactions occurred up to 10,000 bp upstream from the protein-coding genes (Table 4). The analysis of trans-actions between DELs and DEGs revealed the 9295 interaction events occurring with 469 DEGs (assuming a high level of Pearson’s correlation coefficient). Additional analysis of mRNA–lncRNA and protein–lncRNA bindings revealed, respectively, 1393 and 115 cases, restricting the number of previously discovered trans-interactions to 1440 performed with 300 DEGs (Table S1). All detected cis- and trans-interactions between DELs and DEGs are summarized in Figure 4.

### 3.3. Differential Alternative Splicing Events of Differentially Expressed Genes

**Figure 5:**
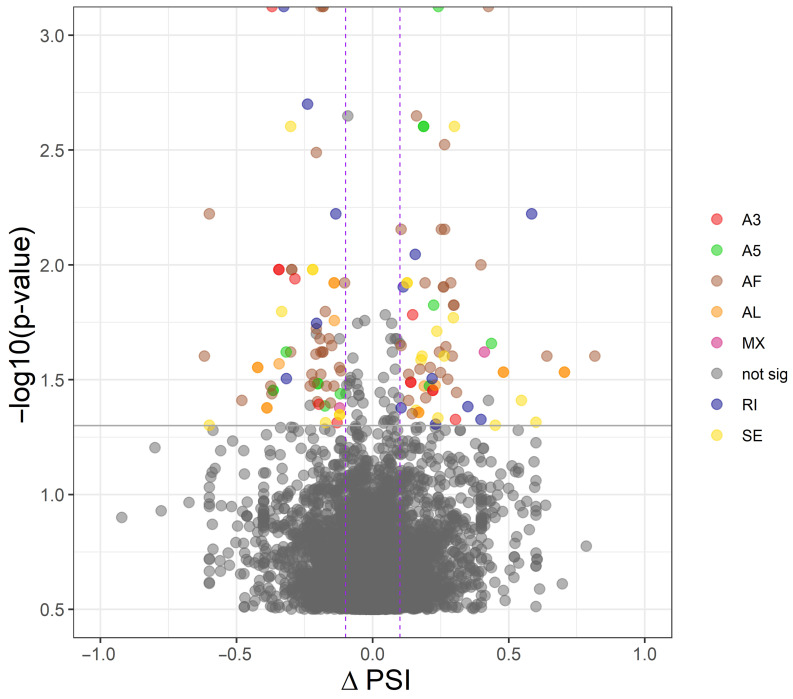
Volcano plot showing the distribution of differential alternative splicing events (DASs). The purple dashed lines and the solid grey line indicate the applied cut-off thresholds, described in detail in the text. Abbreviations: ΔPSI—change of splicing inclusions percentage in chemerin-treated group in relation to control group, A3—alternative 3′ splice site, A5—alternative 5′ splice site, AF—alternative first exon, AL—alternative last exon, MX—mutually exclusive exons, RI—retention intron, SE—skipping exon, not sig—not significant.

**Figure 6:**
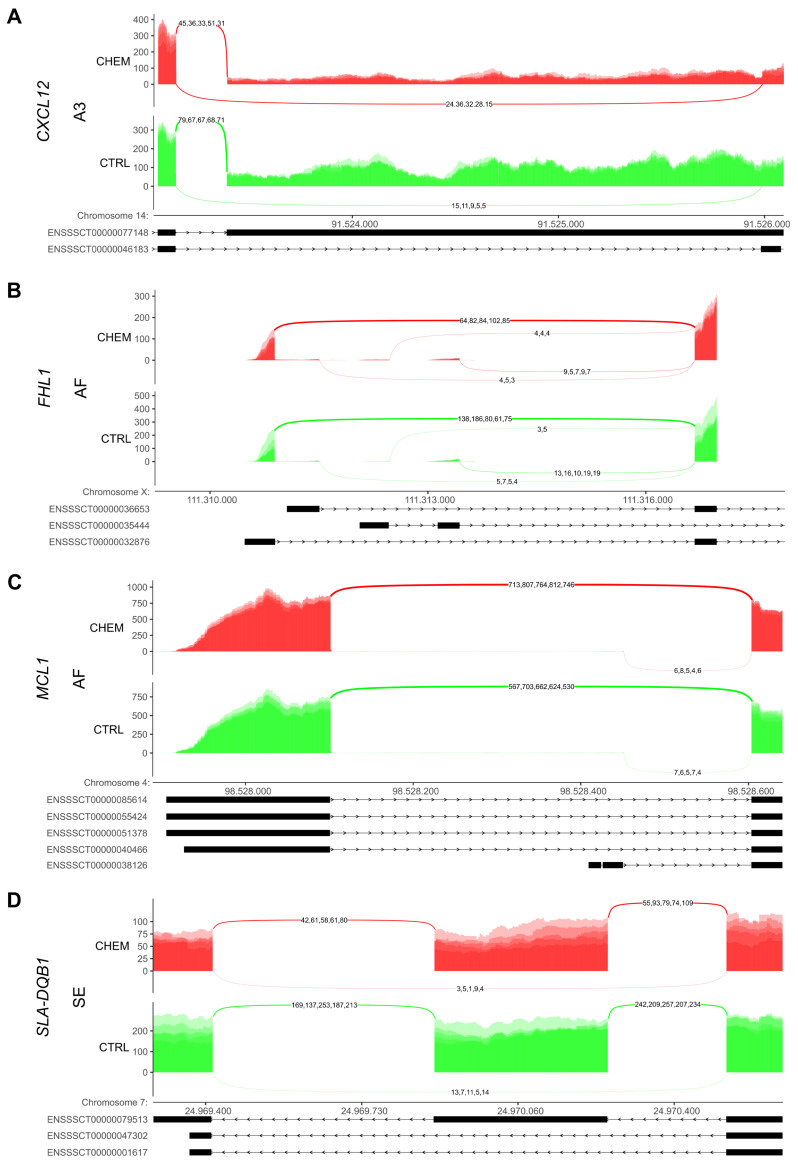
Sashimi plot visualizing the detected coverage of RNA-Seq reads on reference genome, including (**A**) *CXCL12*, (**B**) *FHL1*, (**C**) *MCL1*, and (**D**) *SLA-DQB1* genes fragments classified as statistically significant alternative splicing events. Abbreviations: AF—alternative first exon, A3—alternative 3′ splice site, SE—skipping exon, CHEM—chemerin-treated group, CTRL—control group.

The adopted procedure, incorporating the SUPPA2 tool, allowed the detection of 43,918 alternative splicing events, including 137 DASs resulting from the comparison of CHEM-treated and control samples. All detected DASs are localized within 92 DEGs. Among all detected DASs, 13 were classified as A5, 12 as A3, 2 as MX, 13 as RI, 21 as SE, 60 as AF and 16 as AL (Figure 5). Selected events of alternative splicing occurring within the *CXCL12*, *MCL1*, *FHL1* and *SLA-DQB1* genes are visualized in Figure 6, while all identified cases are summarized in Table S2.

### 3.4. Single Nucleotide Variant Calling and Allele-Specific Expression Variants

**Figure 7:**
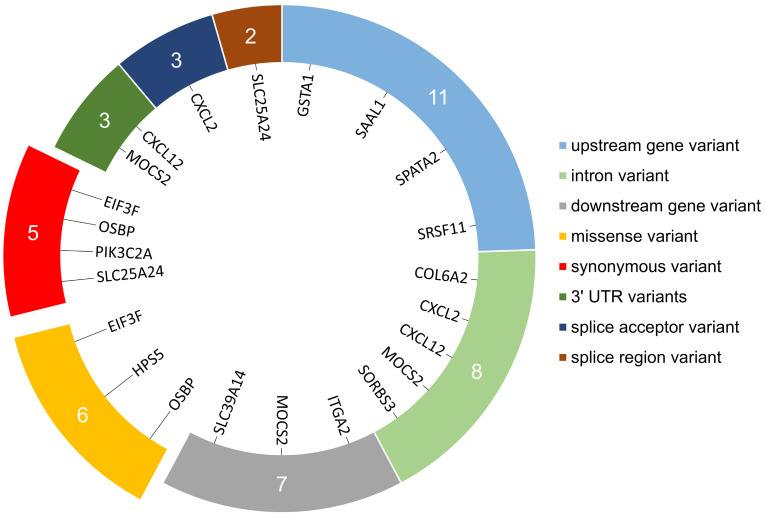
Summary of the location of allele-specific expression variants (ASEs) within each region of target transcripts. Numbers on the coloured ring represent the number of transcripts within which ASEs were identified on specific fragments. The genes encoding the transcripts included in each ASEs location group are listed inside the ring. The protruding blocks represent changes located on the coding DNA sequence (CDS) region. ‘Splice acceptor’ region was defined as the 2 bases at the end of the 3′ intron. ‘Splice region’ is the region at the exon–intron junctions, 1–3 bases at the end of the exon and 3–8 bases at the end of the intron.

During the in silico analyses of ten RNA-Seq libraries, 151,603 SNVs were called for the transcriptome of porcine mid-luteal LCs. After the multi-step filtering procedure, based on the standard GATK parameters and the AAF occurrence in at least half of the samples, 37 SNVs were obtained and directed to the next steps of analysis. Among all remaining SNVs, 18 showed statistically significant allelic imbalance occurring at the same loci (|ΔAAF| > 0.1 and FDR < 0.05) between CHEM-treated and control samples. In accordance with the VEP annotations, 13 ASEs is novel and 5 is previously known: rs1110933145, rs322079801, rs332564681, rs346401196, and rs55619031. Detected ASEs overlapped 38 transcripts encoded by 16 genes. Localization consequences of individual ASEs within the genes and transcripts components were summarized in Figure 7.

As annotated by VEP, 11 ASEs affected non-coding regions of transcripts encoded by 11 genes with consequences difficult to predict. Four ASEs affected CDS regions of transcripts encoded *EIF3F*, *OSBP*, *PIK3C2A* and *SLC25A24* genes with low probability to change coded protein. Four ASEs affected transcripts encoded by *EIF3F*, *HPS5* and *OSBP* genes non-disruptively with chance to change protein effectiveness. Impact of 1 ASE on transcripts encoded by *CXCL2* gene is disruptive and causes loss of protein function by modifying the splice acceptor of second intron. According to the results of the sorting intolerant from tolerant (SIFT) module [91] implemented in VEP, of the 4 missense ASEs that can affect the protein product conformation, 2 have a deleterious effect (on *HPS5* and *OSBP* genes) and 2 could be tolerated (*EIF3F* and *OSBP* genes). The detailed features of discovered ASEs, their biological impact on genes and protein translation process were summarized in Table S3.

### 3.5. Functional Annotation of Target Protein-Coding Genes

In order to summarize and combine the results obtained by analysing three different biological processes occurring at the transcription RNA level, an additional research step was carried out. Initially, the KOBAS tool was used to enrich terms provided in GO, the Reactome Knowledgebase and KEGG databases, separately for each of the three biological phenomena analysed. Gene ontology enrichment analysis of genes cis interacting with DELs, genes trans-interacting with DELs, genes containing DASs and/or ASEs showed a significant contribution of, respectively, 5, 273, 39 and 10 genes encompassed to 30, 159, 2 and 39 biological process (BP) terms, 5, 34, 1 and 17 molecular function (MF) terms, and 4, 24, 3 and 12 cellular component (CC) terms (Table S4). The Reactome Knowledgebase analysis demonstrated the significant enrichment of 3 molecular pathways by genes cis interacting with DELs, 20 pathways by genes trans-interacting with DELs, and 1 pathway by genes containing ASEs (Table S4). The Reactome Knowledgebase enrichment analysis did not identify any statistically significant molecular pathways in which the genes containing DASs were involved.

To predict the detailed biological effect of the observations described in the previous sections, the protein-coding genes related to DELs, DASs and ASEs were distinguished in maps of metabolic or signal transduction KEGG pathways. The 84 enriched pathways indicated the statistical significance. Due to the unrelatedness of the studied tissue and the purpose of this study, the pathways associated with pathological processes, such as autoimmune diseases, carcinogenesis, and infections initiated by bacteria, prions, protists and viruses were not analysed. Details of the remaining 35 pathways associated with physiological mechanisms are summarized in Table 5 and Figures S1–S18. Additionally, important for CLs functioning, “Arachidonic acid metabolism” pathway that did not reach statistical significance during KEGG enrichment was examined (Figure S19).

### 3.6. Quantitative Real-Time PCR Validations

**Figure 8:**
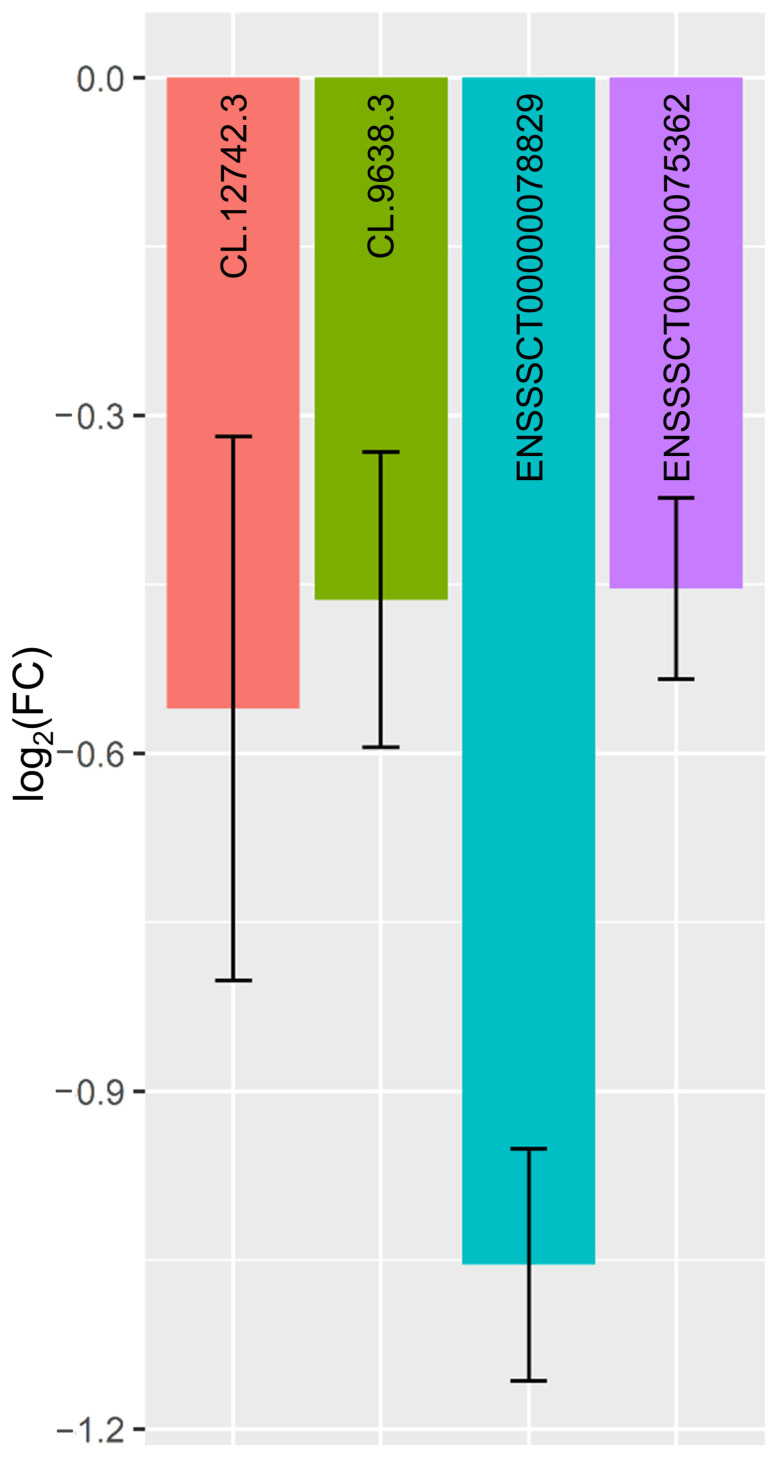
Quantitative real-time PCR validations of RNA-Seq results performed for differentially expressed lncRNAs—*CL.12742.3*, *CL.9638.3*, *ENSSSCT00000078829* and *ENSSSCT00000075362* with reference genes (β-actin and glyceraldehyde-3-phosphate dehydrogenase). All data are expressed as log_2_(FC) ± confidence interval (n = 5). Error bars not crossing the x-axis indicate that the corresponding means comparison is statistically significant to 5% in Student’s *t*-test. Abbreviations: log_2_(FC)—logarithm to base 2 of fold change.

Four DELs were selected for qPCR analysis to validate the obtained RNA-Seq results. The qPCR expression patterns of *CL.9638.3*, *CL.12742.3*, *ENSSSCT00000075362* and *ENSSSCT00000078829* agreed with the RNA-Seq results (Figure 8). Results of qPCR confirmed the accuracy and veracity of the high-throughput methods applied in this study.

## 4. Discussion

This study represented the first ever attempt to describe the CHEM impact on alternative mRNA transcription mechanisms viz. DELs, DASs, and ASEs in the mammalian CLs. The aforementioned analyses were performed on data derived from high-throughput sequencing of the in vitro cultured mid-luteal porcine LCs transcriptomes. Inference of the physiological effects of the molecular processes modified by CHEM was carried out on the basis of interaction or direct association of individual events with protein-coding genes, whose transcriptional profiles were described by Makowczenko et al. [19]. Briefly, we identified 24 DELs that underwent 11 cis interactions with 6 DEGs and 1440 trans-interactions with 300 DEGs, 137 DASs across 92 genes, and 18 ASEs present on transcripts encoded by 16 genes.

One of the three CHEM membrane receptors is CCRL2, often described as an “atypical” receptor due to the lack of chemical signal transduction [92]. Monnier et al. [8] reported a direct association between activation of the MAPK (including NF-κB transcription factors) and Jak/STAT signalling pathways (Figures S5 and S9, respectively) by pro-inflammatory factors and the up-regulation of *CCRL2* gene expression in the human and mouse endothelial cells. In a recent study, we demonstrated the presence of CCRL2 protein in the porcine LCs on days 10–12 of the oestrous cycle [17] and postulated activation of both mentioned signal transduction pathways under the influence of CHEM [19]. Admittedly, we did not note statistically significant differences in *CCRL2* mRNA abundance in CHEM-treated LCs compared to the control group [19]. However, in the current study, we can observe significant differences in the process of alternative splicing, classified as AF with (ΔPSI = −0.48), resulting in increased proportion of *CCRL2* transcript variants containing first exon of 252 nt length in comparison to those containing first exon of 56 nt length. Both alternative sequences contain a fragment of 5′ UTR. It is highly likely that the observed alteration of the first exon may affect the stability of transcriptional variants and/or the translation process, affecting the concentration of CCRL2 proteins on the surface of LCs under the CHEM influence. The effect may promote a chemotactic gradient of CHEM through its binding by CCRL2 proteins, increasing the migration of leukocytes into the CLs, as described in the case of other cell types, such as endothelial and epithelial cells [7,8,93,94]. The above observation requires further detailed studies to elucidate the reported phenomenon.

**Figure 9:**
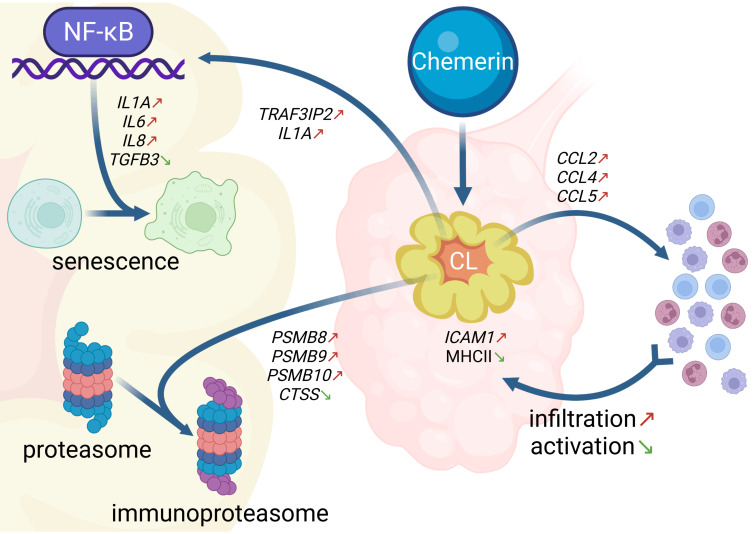
The summary of the CHEM effects on the genes transcription and processes affecting the inflammatory status of porcine CLs during the mid-luteal phase of the oestrous cycle. The arrows next to the processes names or genes abbreviations symbolize the effect of the CHEM influence: ↑ (red)—stimulatory, ↓ (green)—inhibitory. Details are described in the text. Abbreviations: CCL 2/4/5—C-C motif chemokine ligand 2/4/5 genes, CL—corpus luteum, CTSS—cathepsin S gene, ICAM1—intercellular adhesion molecule 1 gene, IL 1A/6/8—interleukin 1A/6/8 genes, MHCII—major histocompatibility complex II, NF-κB—nuclear factor κ-light-chain-enhancer of activated B cells, *PSMB* 8/9/10—proteasome 20S subunit β 8/9/10 genes, *TGFB3*—transforming growth factor β3 gene, *TRAF3IP2*—TNF receptor associated factor 3 interacting protein 2 gene.

Data on the relationship of immune cells and produced by them inflammatory cytokines to the luteolysis management still remain limited in pigs. Nevertheless, according to the hypothesis by Pate and Keyes [95], immune cells are actively involved in the loss of steroidogenic potential and the destruction of CLs. Standaert et al. [96] have shown that in the porcine CLs, immune cells are present in addition to the natively present cells and their number changes depend on the ongoing stage of the oestrous cycle. Ziecik et al. [97] have shown that genes related to lymphocyte infiltration are activated in CLs with the onset of functional luteal regression in gilts. Moreover, increased monocytes/macrophages abundance during the late-luteal phase is directly related to the presence of pro-inflammatory cytokines that regulate CLs apoptosis and structural luteolysis. In our previous study, we have suggested that CHEM may indirectly regulate the process of luteolysis by influencing the immune system cells motility as the chemotactic factor and agent that modifies the levels of pro- and anti-inflammatory cytokines [17]. Among the data analysed, we noted a significant effect of DELs and ASEs on genes involved in TNF signalling pathway (Figure S2), the activation of which results in increased production of the cytokines, chemokines, and adhesion factors analysed in the following paragraphs. The CHEM effects on the genes transcription and processes affecting the inflammatory status of porcine CLs during the mid-luteal phase of the oestrous cycle are summarized in Figure 9.

The chemokines CCL2 and CCL4 are known as chemoattractants and activators of immune cells, such as monocytes, T cells, and NK cells [98,99]. The main function of CCL5 is to activate diverse lymphocyte populations, but it can also stimulate eosinophils, basophils, and dendritic cells [100]. Witek et al. [101] in a recent study on the porcine CLs found that expression of genes encoding CCL2, CCL4, and CCL5 increases during the late-luteal phase of the oestrous cycle compared to the mid-luteal phase. During our previous study [19], we found that the concentration of *CCL2*, *CCL4*, and *CCL5* mRNAs increased in response to CHEM. The current study revealed the negative correlations of these genes’ transcriptional profiles with DELs’ profiles. We have also identified this DEGs’ intermolecular bonding with DELs at the mRNA–lncRNA level (one for *CCL2* and *CCL5* each), and protein–lncRNA level (2 for *CCL2* and 11 for *CCL4*). The presence of CHEM in the environment, via effects on genes encoding the described chemokines, may contribute to leukocyte infiltration into CLs. In situ, based on additional signals from LCs, immune cells may initiate luteal regression.

This thesis is also supported by the increase in the levels of *IL1A* and *TRAF3IP2* mRNAs [19] and their interactions at the protein–lncRNA level with DELs (6 actions per gene). Both genes are involved in the expression induction of, among others, *IL1A*, *IL6*, *IL8* (also named *CXCL8*) and *TGFB3*, as the result of the NF-κB signal transduction pathway activation (Figure S15). Under CHEM influence, the mRNAs abundance of these interleukins was strongly up-regulated, whereas the transcription of *TGFB3* down-regulated [19]. We identified protein–lncRNA interactions with DELs for each of the four genes (6, 3, 3, and 2 trans-actions respectively). The expression profiles of these factors are distinctive of cellular senescence [102,103]. The senescent cells can potentially attract and activate different immune cells subsets (such as NK cells, B cells, T cells, neutrophils, dendritic cells or monocytes/macrophages), which in turn, can be engaged in the immune response and removal of senescent cells [104]. Interestingly, two research teams reported *ICAM1* expression up-regulation in the senescent cells [105,106], which may facilitate communication with immune cells and induce luteal regression, as described by Olson et al. in rats [107]. In our previous studies, *ICAM1* mRNA concentration was up-regulated [19]. Furthermore, 6 trans-interactions with DELs at protein–lncRNA level were identified.

Among all chemokines’ gene transcriptional profiles analysed in this and previous work [19], only the transcription of *CXCL12* (encoding SDF-1 chemokine protein) decreased, with a simultaneous increase in the mRNA abundance of its receptor—*CXCR4*. Within *CXCL12*, we identified DAS event classified as A3 (ΔPSI = −0.37), resulting in the formation of transcripts lacking 2597 nt fragment within the penultimate exon containing CDS-ending sequence and partial 3′ UTR sequence (Figure 6A). Additionally, in CHEM-treated cells we identified an ASE site numbered rs346401196 in the Ensembl database (ΔAAF = 0.187), resulting in an increase in the frequency of nucleotide C, at site A within the last exon containing a partial 3′ UTR sequence (or intron in some transcript variants; Figure 7). Expression of *CXCL12* and *CXCR4* genes was found in the human pre-ovulatory GCs [108] as well as in the sheep LCs [109,110]. Available reports focus on SDF-1 and CXCR4 proteins contribution to chemotactic activity of immune cells and the resulting consequences for folliculogenesis [108,111,112]. Although little is known about these proteins’ involvement in CLs regulation, McIntosh et al. [110] observed the increased expression of both genes in LCs during early stages of pregnancy. The researchers indicated the luteotropic effect of SDF-1 conductive to the establishment and maintenance of intrauterine environment suitable for conceptus implantation. Furthermore, Nishigaki et al. hypothesized that SDF-1 may facilitate luteinization of human preovulatory follicles [113]. The potential changes in the amount of SDF-1 and CXCR4 proteins in CHEM-treated LCs may suggest CHEM influence on the chemokine action and, indirectly, leukocytes infiltration of the luteal tissue. This phenomenon may be related to the discovery of de Poorter et al. [114] regarding the possibility of heteromer formation between the CHEM receptor CMKLR1 and CXCR4. This may have important implications for the transduction of signals by both types of receptors, mainly cross-inhibition [3]. Furthermore, the same research team found that CHEM co-incubated with SDF-1 decreases their specific binding to mouse bone marrow-derived dendritic cells [114]. The SDF-1 and CXCR4 protein pair is involved in a number of molecular processes, such as “Cytokine-cytokine receptor interaction” (Figure S1), “NF-kappa B signalling pathway” (Figure S5), “Regulation of actin cytoskeleton” (Figure S13), and ‘Leukocyte transendothelial migration’ (Figure S14).

Noteworthy are the results obtained during our previous research [19], in which the abundance of the *HLA-DRA*, *SLA-DMA*, and *SLA-DQB1* mRNAs decreased under CHEM treatment in the swine mid-luteal LCs. Furthermore, we detected 4 (2 positively and 2 negatively corelated), 1 (positively corelated), and 8 (7 positively and 1 negatively corelated) trans-interactions of mentioned genes with DELs, respectively, and DAS event qualified as SE within the *SLA-DQB1* gene, resulting in the absence of exon 4 in the final transcripts (ΔPSI = 0.26; Figure 6D). The proteins encoded by these genes are antigens belonging to the MHC II group [115]. Members of this group are present on the small and large LCs, and their expression increases significantly after the onset of luteal regression in humans, cattle and ewes [116,117,118]. MHC II class peptides are recognized by CD4+ T cells (Figure S10), activation, of which results in their clonal expansion and leads to the release of cytokines such as TNFα and IFNγ [95]. Therefore, when the expression of these genes is inhibited and/or the formation of shorter length transcript variants is promoted, the amount of MHC II membrane proteins is also reduced. This may lead to suppressed CD4+ T lymphocytes activation and inhibition of secretion of luteolytic cytokines into the CLs microenvironment.

On the other hand, all three genes—*PSMB9*, *PSMB10*, *PSMB8*, encoding the β1i, β2i, and β5i particles of the immunoproteasome 20S catalytic subunit, respectively, and *PSME1* and *PSME2* genes encoding two particles, PA28α and PA28β, building the immunoproteasome 19S regulatory subunit in cytosol [119], were all up-regulated in CHEM-treated swine LCs [19]. Additionally, for *PSMB10* (1 positively correlated), *PSMB8* (4 negatively correlated)*,* and *PSME2* (1 positively and 3 negatively correlated), *trans*-interactions with DELs were detected (Figure S17). Immunoproteasomes degrade ubiquitin-tagged proteins by preparing the hydrophobic C-ends of the proteins to fit into the grooves of MHC I complexes (Figure S10), allowing the prepared fragments to be exposed to CD8+ T lymphocytes which may lead to cell destruction (apoptosis) through a cytotoxic effect [119]. The expression of proteins building immunoproteasomes can significantly increase in cells exposed to pro-inflammatory cytokines, or oxidative stressors [119,120]. Most notably, Luo and Wiltbank [121] reported increased expression of *PSMB9*, *PSMB8*, *PSMB10*, and *CTSS* genes in the luteinized bovine GCs co-cultured with T cells. Interestingly, Cash et al. [122] demonstrated the conversion of CHEM molecules into shorter fragments with potent anti-inflammatory effects by a number of cysteine proteases, including CTSS. During the current study, we identified two cases of DASs in the *CTSS* gene, classified as an AF (ΔPSI = −0.30 and −0.18) which may result in modification of the mature protein. The presented modifications in the expression of genes which products build immunoproteasomes, as well as interactions with DELs and the occurrence of DASs in the porcine mid-luteal LCs, would suggest the involvement of CHEM in cellular response that can lead to degradation of CLs.

Among the obtained functional analysis results, we observe the high proportion of signalling pathways and metabolic processes directly related to immune responses, such as “IL-17 signalling pathway” (Figure S3), “NOD-like receptor signalling pathway” (Figure S4), “Toll-like receptor signalling pathway” (Figure S7), “Cell adhesion molecules (CAMs)” (Figure S11), and ‘Phagosome’ (Figure S18). This is further evidence of the CHEM influence on the intensification of processes related to immune mechanisms in the microenvironment of the porcine mid-luteal CLs. The limitation of the number of immune cells in in vitro cell culture research models makes it impossible to observe all the nuances of the reported molecular processes that occur in living organisms, and further studies to shed new light on the mentioned phenomena are worthwhile.

Within *FHL1*, the gene linked to the Jak/STAT signal transduction pathway, we found five forms of statistically significant DASs categorized as AF, whose occurrence was caused by CHEM (ΔPSI ∈ {−0.22} ∪ [0.13, 0.30]; Figure 6B). The biological function of the protein encoded by this gene is poorly understood in the processes unrelated to carcinogenesis. However, Fimia et al. [123] demonstrated expression of *FHL1* in the mammalian ovarian cells. Matulis & Mayo [124] linked the knock-down of this gene to the down-regulation of the NR5A transcription factor in the mouse GCs, which directly affects the production of proteins important for steroidogenesis, such as CYP11A1 (also referred to as P450scc) catalysing the conversion of cholesterol to pregnenolone, the direct substrate of progesterone. Also, accordingly to Ziecik et al. [97], expression of *NR5A1* gene was down-regulated on day 14 vs. day 12 (late-luteal vs. mid-luteal phase) of the estrous cycle in porcine CLs, which may be associated with the acquisition of luteolytic sensitivity. The appearance of DASs within *FHL1* affecting the decreased activity of the proteins produced would explain the decrease in *CYP11A1* mRNA concentration and its protein production under the CHEM influence in ovarian cells [10].

We can infer a positive effect of CHEM on angiogenesis in the mid-luteal CLs of gilts based on observations both at the transcriptomic level—increase in *VEGFA* mRNA level [19], negative correlation with seven DELs showing six protein–lncRNA interactions and one mRNA–lncRNA interaction, and at the protein level—increase in VEGFA secretion [21]. Vascular endothelial growth factor A through its signal transduction pathway (Figure S16), plays a major role in endothelial cell survival, vascular stabilization and integrity [125,126,127]. This finding seems to be in line with CHEM effect on PGE_2_ production. By treating the porcine mid-luteal LCs with CHEM, we observed an increase in mRNA abundance of *PTGS2* and *PTGES* creating the pathway from the primary substrate—arachidonic acid to the production of luteotropic factor—PGE_2_ [19]. Moreover, we have found the protein–lncRNA *trans*-interactions associated with negative correlation of this DEGs’ transcriptional profiles with 3 and 2 DELs, respectively (Figure S19). According to the “two signal switch” hypothesis, PGE_2_, acting on LCs around day 12 of the oestrous cycle in pigs, prevents the acquisition of luteolytic sensitivity by activating the so-called “rescue switch” that results in the maintenance of angiogenesis, steroidogenesis, and LCs’ survival [97].

In previous studies, we have reported a stimulating effect of CHEM on concentration of *CASP3* mRNA encoding the major execution protease of the apoptosis process in the porcine LCs [19]. During the present study, we identified the lncRNA molecule *CL.9638.3*. Its expression was inhibited by CHEM and was negatively correlated with that of *CASP3* (Figure S12). For both molecules, we additionally detected the probability of combining protein–lncRNA spatial structures of 94.66%. However, it should be noted that the recent study by our team, in which the ELISA method was implemented, showed no effect of CHEM on the amount of CASP3 protein produced by the mid-luteal LCs of gilts [21]. This allows us to assume that in spite of CHEM influence on CASP3 generation at the transcriptomic level, this effect is neutralized during the next steps of its production. However, it cannot be ruled out that CHEM may modify CASP3 protein changing its activity. The evidence of the CHEM influence on the process of apoptosis was the increased expression of two lncRNAs—*CL.9638.3* and *CL.2666.5*—showing high probability of binding to MCL1 proteins (95.59% and 90.56%, respectively) and exhibiting significant correlation values of transcription levels (r = −0.97 and 0.96). Furthermore, within the *MCL1* gene, we identified DAS event, classified as AF (ΔPSI = −0.18; Figure 6C). In the previous publication [19] we demonstrated the *MCL1* mRNA abundance under the CHEM-treatment increased. Interestingly, Shee-Uan et al. [128] found that the increase in *MCL1* expression in LCs prevents apoptosis and increases cell viability, and thus may play an important role in regulating the lifespan of CLs. It seems, therefore, that CHEM effect on MCL1 production and apoptosis process in porcine CLs is ambiguous. It is also worth paying attention to the slight increase in the percentage of splicing inclusions classified as RI event within *DUSP4* (ΔPSI = 0.11), whose transcription enhancement under the influence of CHEM we previously observed [19]. The aforementioned CHEM effect consisted in generation suppression of transcript variants containing the last exon with the terminal CDS fragment and 3′ UTR at the advantage of the variant having two exons encoding analogous regions of the transcript. The *DUSP4* encodes the nuclear phosphatase involved in ERK and p38 dephosphorylation (Figure S6), and thereby controlling the activity of the MAPK–ERK1/2–p38 signal transduction pathway [129,130]. Increased expression of *DUSP4* results in providing an anti-apoptotic effect and promotes cell survival [131,132].

An interesting observation that requires further detailed study seems to be the significant effect of CHEM on genes involved in necroptosis (a caspase-independent form of programmed cell death reminiscent of necrosis) in the mid-luteal LCs of gilts (Figure S8). Hojo et al. [133], studying bovine CLs, concluded that luteolysis is an acute tissue damage process, whereas the mechanism of apoptosis itself is not acute. It seems that an efficient regression of the CLs must consist of a number of other processes, including necroptosis. Markers of necroptosis are the proteins RIPK1 and RIPK3, whose activation increases during luteal regression and which, forming homo- and heteromers, phosphorylate a number of proteins that are necroptosis executors [133,134]. No differences in the mRNA abundance and occurrence of transcription-associated phenomena (DELs interactions, DASs and ASEs) were found for any gene encoding the aforementioned proteins [19]. Nonetheless, we observed the increase in the transcription of some genes and an intensification of transcription-associated mechanisms in genes involved in the signal transduction pathways, that are also involved in both ubiquitination (TNF pathway; Figure S2) and induction of *RIPK1* expression (Jak/STAT pathway; Figure S9). Moreover, CHEM increased *TNFAIP3* mRNA concentration [19] and revealed 6 negatively correlated interactions with DELs at the protein–lncRNA level. The protein encoded by this gene shows the ability to deubiquitinate the other pro-necroptotic factor, RIPK3. These finding indicate the possibility of CHEM-induced specific preparation of cells for necroptosis which could be triggered by additional factors.

## 5. Conclusions

Performing in silico analyses of alternative mRNA transcription related to CHEM effects on the porcine LCs enabled a more detailed insight and description of the sophisticated and subtle molecular mechanisms controlling cells functions. We have gained further arguments indicating that CHEM affects the MAPK (including NF-κB transcription factors) and Jak/STAT signalling pathways, local generation and action of cytokines and chemokines, as well as essential for CLs processes such as angiogenesis, prostaglandins production and steroidogenesis. The phenomena analysed at the molecular level indicate activation of both pro-apoptotic (or even pro-necroptotic) and pro-survival mechanisms, supporting CHEM engagement in the control of CLs lifespan.

## Figures and Tables

**Table 2:** Summary of mapping of reads to the porcine reference genome. All numerical values are expressed in millions.

Treatment	CTRL					CHEM				
Samples	1_LC	2_LC	3_LC	4_LC	5_LC	1_LC	2_LC	3_LC	4_LC	5_LC
Mapped reads	97.380	111.081	106.721	102.366	95.792	97.190	102.650	99.617	108.891	100.253
Uniquely mapped reads	94.544	106.781	103.010	98.833	92.527	93.573	99.472	95.824	104.621	96.646
% of uniquely mapped reads	96.86%	95.89%	96.30%	96.32%	96.37%	96.06%	96.67%	95.96%	95.84%	96.17%
Multi-mapped reads	2.482	3.968	3.314	3.136	2.973	3.312	2.736	3.489	3.934	3.274
% of bases mapped to CDS	60.70%	58.88%	59.83%	59.31%	60.05%	59.22%	59.38%	58.87%	59.15%	59.83%
% of bases mapped to UTR	22.89%	23.44%	23.09%	23.50%	23.16%	23.29%	23.91%	23.79%	23.13%	22.97%
% of bases mapped to introns	3.31%	3.21%	3.35%	3.43%	3.21%	3.49%	3.32%	3.25%	3.47%	3.43%
% of bases mapped to intergenic	13.10%	14.48%	13.73%	13.76%	13.59%	14.00%	13.38%	14.08%	14.24%	13.76%

Abbreviations: CDS—coding DNA sequence regions, CHEM—chemerin-treated group, CTRL—control group, UTR—untranslated region.

**Table 3:** Differentially expressed lncRNAs discovered in the porcine luteal cells treated with chemerin during the mid-luteal phase of the oestrous cycle.

Transcript ID	Reference Gene ID	log_2_(FC)	*q*-Value	Regulation	Chr	Str	Start–End
ENSSSCT00000076362	ENSSSCG00000047565	2.17	1.39 × 10^−3^	up	18	−	42,660,099–42,669,101
CL.2666.1	N/A	1.49	2.03 × 10^−4^	up	12	+	40,817,836–40,834,552
CL.2666.5	N/A	1.49	2.03 × 10^−4^	up	12	+	40,817,901–40,850,478
ENSSSCT00000090963	ENSSSCG00000050550	1.48	2.85 × 10^−6^	up	11	+	19,098,312–19,102,389
ENSSSCT00000066632	ENSSSCG00000042788	1.11	1.92 × 10^−2^	up	5	−	5,752,685–5,756,310
ENSSSCT00000068213	ENSSSCG00000042788	1.11	1.92 × 10^−2^	up	5	−	5,752,686–5,770,732
ENSSSCT00000028661	ENSSSCG00000028322	0.67	1.08 × 10^−4^	up	9	+	64,031,854–64,037,769
ENSSSCT00000068487	ENSSSCG00000050423	0.54	1.69 × 10^−4^	up	13	+	21,429,679–21,433,475
CL.12742.3	ENSSSCG00000036505	−0.58	2.29 × 10^−5^	down	6	+	144,782,442–144,918,548
ENSSSCT00000038686	ENSSSCG00000036505	−0.58	2.29 × 10^−5^	down	6	+	144,782,503–144,918,549
ENSSSCT00000046229	ENSSSCG00000036505	−0.58	2.29 × 10^−5^	down	6	+	144,782,552–144,918,549
ENSSSCT00000047711	ENSSSCG00000036505	−0.58	2.29 × 10^−5^	down	6	+	144,782,576–144,918,548
ENSSSCT00000076188	ENSSSCG00000036505	−0.58	2.29 × 10^−5^	down	6	+	144,823,167–144,918,548
ENSSSCT00000087136	ENSSSCG00000036505	−0.58	2.29 × 10^−5^	down	6	+	144,781,500–144,912,714
CL.9638.3	N/A	−0.71	8.77 × 10^−4^	down	4	−	8,075,901–8,081,556
ENSSSCT00000036447	ENSSSCG00000006581	−0.71	7.46 × 10^−3^	down	4	+	96,035,969–96,037,483
ENSSSCT00000086415	ENSSSCG00000048436	−0.85	4.04 × 10^−2^	down	12	−	20,101,333–20,108,070
ENSSSCT00000077934	ENSSSCG00000050649	−0.99	2.82 × 10^−4^	down	2	−	1,381,838–1,383,221
ENSSSCT00000078829	ENSSSCG00000050649	−0.99	2.82 × 10^−4^	down	2	−	1,381,841–1,384,446
ENSSSCT00000081255	ENSSSCG00000050649	−0.99	2.82 × 10^−4^	down	2	−	1,381,839–1,383,160
ENSSSCT00000084501	ENSSSCG00000050649	−0.99	2.82 × 10^−4^	down	2	−	1,381,841–1,384,446
ENSSSCT00000072909	ENSSSCG00000048033	−1.82	7.70 × 10^−4^	down	3	−	112,308,405–112,318,405
ENSSSCT00000075362	ENSSSCG00000048033	−1.82	7.70 × 10^−4^	down	3	−	112,308,405–112,318,340
ENSSSCT00000080610	ENSSSCG00000048033	−1.82	7.70 × 10^−4^	down	3	−	112,308,405–112,318,450

Abbreviations: Chr—chromosome number, CL—Stringtie’s gene identifier, log2(FC)—logarithm to base 2 of fold change, N/A—not available, Str—strand, ‘+’—sense strand, ‘−’—antisense strand.

**Table 4:** Summary of cis-interactions between chemerin-induced differentially expressed long non-coding RNAs and differentially expressed genes identified in the porcine luteal cells during days 10–12 of the oestrous cycle.

lncRNA ID	Partner mRNA ID	Partner Gene Name	Dir	Type	Distance	Location
CL.9638.3	ENSSSCT00000006529	*CCN4*	+	genic—overlapping	0	exonic
CL.9638.3	ENSSSCT00000060888	*CCN4*	+	genic—overlapping	0	exonic
CL.9638.3	ENSSSCT00000047671	*CCN4*	+	genic—overlapping	0	exonic
ENSSSCT00000080610	ENSSSCT00000009368	*KCNK3*	+	intergenic—same_strand	3996	downstream
ENSSSCT00000072909	ENSSSCT00000009368	*KCNK3*	+	intergenic—same_strand	4041	downstream
ENSSSCT00000075362	ENSSSCT00000009368	*KCNK3*	+	intergenic—same_strand	4106	downstream
ENSSSCT00000036447	ENSSSCT00000042276	ENSSSCG00000038991	+	intergenic—same_strand	6100	downstream
ENSSSCT00000036447	ENSSSCT00000007211	*S100A14*	+	intergenic—same_strand	6100	downstream
ENSSSCT00000086415	ENSSSCT00000076358	*RAMP2*	+	intergenic—same_strand	6859	upstream
ENSSSCT00000086415	ENSSSCT00000018929	*RAMP2*	+	intergenic—same_strand	6864	upstream
ENSSSCT00000076362	ENSSSCT00000048117	*ZNRF2*	+	intergenic—same_strand	8292	upstream

Abbreviations: CL—Stringtie’s gene identifier, Dir—direction, ‘+’—the same strand.

**Table 5:** Summary of KEGG pathways that are statistically implicated by protein-coding genes associated with DELs, DASs, and ASEs occurring in the chemerin-treated porcine luteal cells during days 10–12 of the oestrous cycle. The ‘Visualization’ column contains the numbers of figures available in the supplemental materials presenting molecular pathways included in the discussion.

Pathway Name	Pathway ID	Input (Background) Number	FDR	Visualization
Cytokine-cytokine receptor interaction	ssc04060	25 (259)	4.74 × 10^−11^	Figure S1
TNF signalling pathway	ssc04668	17 (107)	2.94 × 10^−10^	Figure S2
IL-17 signalling pathway	ssc04657	15 (88)	1.96 × 10^−9^	Figure S3
C-type lectin receptor signalling pathway	ssc04625	15 (103)	1.13 × 10^−8^	-
NOD-like receptor signalling pathway	ssc04621	17 (148)	1.56 × 10^−8^	Figure S4
NF-kappa B signalling pathway	ssc04064	13 (99)	3.89 × 10^−7^	Figure S5
Focal adhesion	ssc04510	16 (194)	1.98 × 10^−6^	-
MAPK signalling pathway	ssc04010	19 (286)	2.43 × 10^−6^	Figure S6
PI3K-Akt signalling pathway	ssc04151	20 (335)	4.28 × 10^−6^	-
Hematopoietic cell lineage	ssc04640	11 (88)	4.93 × 10^−6^	-
Toll-like receptor signalling pathway	ssc04620	11 (95)	8.69 × 10^−6^	Figure S7
Necroptosis	ssc04217	13 (147)	1.11 × 10^−5^	Figure S8
Jak-STAT signalling pathway	ssc04630	13 (151)	1.41 × 10^−5^	Figure S9
ECM-receptor interaction	ssc04512	10 (84)	2.08 × 10^−5^	-
Antigen processing and presentation	ssc04612	9 (45)	3.78 × 10^−5^	Figure S10
Cell adhesion molecules (CAMs)	ssc04514	12 (143)	4.46 × 10^−5^	Figure S11
Th17 cell differentiation	ssc04659	10 (107)	1.20 × 10^−4^	-
Apoptosis	ssc04210	11 (134)	1.23 × 10^−4^	Figure S12
Chemokine signalling pathway	ssc04062	11 (177)	1.03 × 10^−3^	-
Th1 and Th2 cell differentiation	ssc04658	8 (91)	1.18 × 10^−3^	-
Regulation of actin cytoskeleton	ssc04810	11 (207)	3.04 × 10^−3^	Figure S13
Leukocyte transendothelial migration	ssc04670	8 (110)	3.21 × 10^−3^	Figure S14
Phospholipase D signalling pathway	ssc04072	9 (146)	4.00 × 10^−3^	-
Cellular senescence	ssc04218	9 (154)	5.43 × 10^−3^	Figure S15
HIF-1 signalling pathway	ssc04066	7 (108)	1.10 × 10^−2^	-
Cytosolic DNA-sensing pathway	ssc04623	5 (55)	1.39 × 10^−2^	-
VEGF signalling pathway	ssc04370	5 (55)	1.39 × 10^−2^	Figure S16
RIG-I-like receptor signalling pathway	ssc04622	5 (63)	2.08 × 10^−2^	-
Rap1 signalling pathway	ssc04015	9 (211)	2.59 × 10^−2^	-
Adipocytokine signalling pathway	ssc04920	5 (71)	2.97 × 10^−2^	-
Proteasome	ssc03050	4 (44)	3.11 × 10^−2^	Figure S17
Ras signalling pathway	ssc04014	9 (229)	3.53 × 10^−2^	-
Phagosome	ssc04145	8 (126)	3.87 × 10^−2^	Figure S18
Folate biosynthesis	ssc00790	3 (24)	3.92 × 10^−2^	-

Abbreviations: ASEs—allele-specific expression variants, DASs—Differential alternative splicing events, DELs—differentially expressed lncRNAs, FDR—false discovery rate.

## Data Availability

Details of the experimental procedures, raw reads and expression data obtained from all of control and chemerin-treated RNA-Seq libraries were deposited in the European Nucleotide Archive database under accession number PRJEB35892 (https://www.ebi.ac.uk/ena/browser/view/PRJEB35892 (accessed on 14 February 2022)) and ArrayExpress Archive of Functional Genomics Data under accession number E-MTAB-11434 (https://www.ebi.ac.uk/arrayexpress/experiments/E-MTAB-11434/ (accessed on 14 February 2022)).

## Supplementary Materials

The following are available online at https://www.mdpi.com/article/10.3390/cells11040715/s1, Table S1: Overview of *trans*-interactions between DELs and DEGs, Table S2: Overview of detected DASs, Table S3: Overview of detected ASEs, Table S4: Results of GO and Reactome functional analysis of DEGs acting with DELs or within which DASs or ASEs were identified, Figure S1: Cytokine-cytokine receptor interaction with DELs’ interactions, DASs and ASEs mapped, Figure S2: TNF signalling pathway with DELs’ interactions and ASEs mapped, Figure S3: IL-17 signalling pathway with DELs’ interactions, DASs and ASEs mapped, Figure S4: NOD-like receptor signalling pathway with DELs’ interactions and ASEs mapped, Figure S5: NF-kappa B signalling pathway with DELs’ interactions, DASs and ASEs mapped, Figure S6: MAPK signalling pathway with DELs’ interactions and DASs mapped, Figure S7: Toll-like receptor signalling pathway with DELs’ interactions mapped, Figure S8: Necroptosis with DELs’ interactions and ASEs mapped, Figure S9: Jak-STAT signalling pathway with DELs’ interactions and DASs mapped, Figure S10: Antigen processing and presentation with DELs’ interactions and DASs mapped, Figure S11: Cell adhesion molecules (CAMs) with DELs’ interactions and DASs mapped, Figure S12: Apoptosis with DELs’ interactions and DASs mapped, Figure S13: Regulation of actin cytoskeleton with DELs’ interactions, DASs and ASEs mapped, Figure S14: Leukocyte transendothelial migration with DELs’ interactions, DASs and ASEs mapped, Figure S15: Cellular senescence with DELs’ interactions mapped, Figure S16: VEGF signalling pathway with DELs’ interactions mapped, Figure S17: Proteasome with DELs’ interactions mapped, Figure S18: Phagosome with DELs’ interactions, DASs and ASEs mapped, Figure S19: Arachidonic acid metabolism with DELs’ interactions mapped.

## Abbreviations

ACTB	Β-actin
Akt	Protein kinase b
AMPK	5′ adenosine monophosphate-activated protein kinase
CASP3	Caspase 3
CCL*	C-C motif chemokine ligand *(2, 4, 5)
CCRL2	C-C chemokine receptor-like 2
ChemR23	Chemerin receptor 23
CMKLR1	Chemerin Chemokine-Like Receptor 1
COL6A2	Collagen type VI α2 chain
CTSS	Cathepsin S
CXCL*	C-X-C motif chemokine ligand *(2, 8, 12)
CXCR4	C-X-C motif chemokine receptor 4
CYP11A1	Cytochrome P450 family 11 subfamily A member 1
DUSP4	Dual specificity phosphatase 4
EIF3F	Eukaryotic translation initiation factor 3 subunit F
ERK*	Extracellular signal-regulated kinase *(1/2)
FHL1	Four and a half LIM domains 1
GAPDH	Glyceraldehyde-3-phosphate dehydrogenase
GPR1	G protein-coupled receptor 1
GSTA1	Glutathione S-transferase α1
HLA-DRA	HLA class II histocompatibility antigen, DRα
HPS5	HPS5 biogenesis of lysosomal organelles complex 2 subunit 2
ICAM1	Intercellular adhesion molecule 1
IFNγ	Interferon γ
IL*	Interleukin *(1A, 6, 8, 17)
ITGA2	Integrin subunit α2
Jak	Janus kinase
MAPK	Mitogen-activated protein kinase
MCL1	Mcl1 apoptosis regulator, bcl2 family member
MHC*	Major histocompatibility complex *(I, II)
MOCS2	Molybdenum cofactor synthesis 2
NF-κB	Nuclear factor κ-light-chain-enhancer of activated B cells
NR5A*	Nuclear receptor subfamily 5 group A *(1)
OSBP	Oxysterol binding protein
p38	Mitogen-activated protein kinase 14
P450scc	Cytochrome P450, subfamily XIA (cholesterol side chain cleavage)
PA28*	Proteasome activator *(α, β)
PBS	Phosphate-buffered saline
PGE_2_	Prostaglandin E_2_
PI3K	Phosphoinositide 3-kinases
PIK3C2A	Phosphatidylinositol-4-phosphate 3-kinase catalytic subunit type 2α
PSMB*	Proteasome 20S subunit β *(8, 9, 10)
PSME*	Proteasome activator subunit *(1, 2)
PTGES	Prostaglandin E synthase
PTGS2	Prostaglandin-endoperoxide synthase 2
RARRES2	Retinoic acid receptor responder 2
RIPK*	Receptor interacting serine/threonine kinase *(1, 3)
SAAL1	Serum amyloid A like 1
SDF-1	Stromal cell-derived factor 1
SLA-DMA	SLA class II histocompatibility antigen, DMα
SLA-DQB1	SLA class II histocompatibility antigen, DQβ1
SLC25A24	Solute carrier family 25 member 24
SLC39A14	Solute carrier family 39 member 14
SORBS3	Sorbin and SH3 domain containing 3
SPATA2	Spermatogenesis associated 2
SRSF11	Serine and arginine rich splicing factor 11
STAT	Signal transducer and activator of transcription
TGFB3	Transforming growth factor β3
TIG2	Tazarotene-induced gene 2
TNF*	Tumour necrosis factor *(α)
TNFAIP3	TNF α induced protein 3
TRAF3IP2	TNF receptor associated factor 3 interacting protein 2
VEGFA	Vascular endothelial growth factor A
