# Supplementary figures and images for: Chemerin Impact on Alternative mRNA Transcription in the Porcine Luteal Cells
Source: Cells. 2022 Feb 17;11(4):715. doi: 10.3390/cells11040715 (PMC8870241; doi:10.3390/cells11040715)

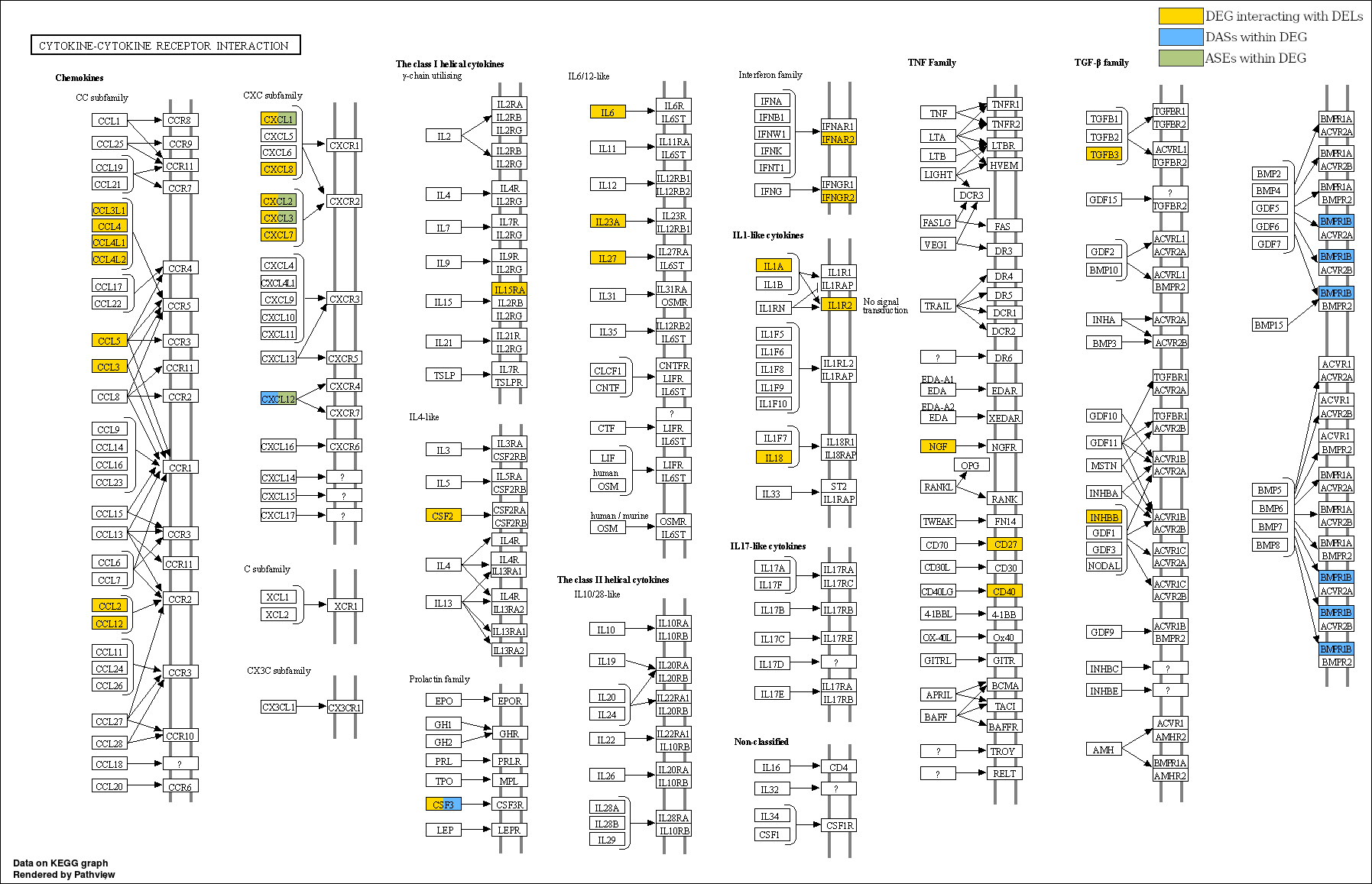

Supplement: Supplementary file 1 [file cells-11-00715-s001.zip › Supplementary_materials/Figure_S1.png]

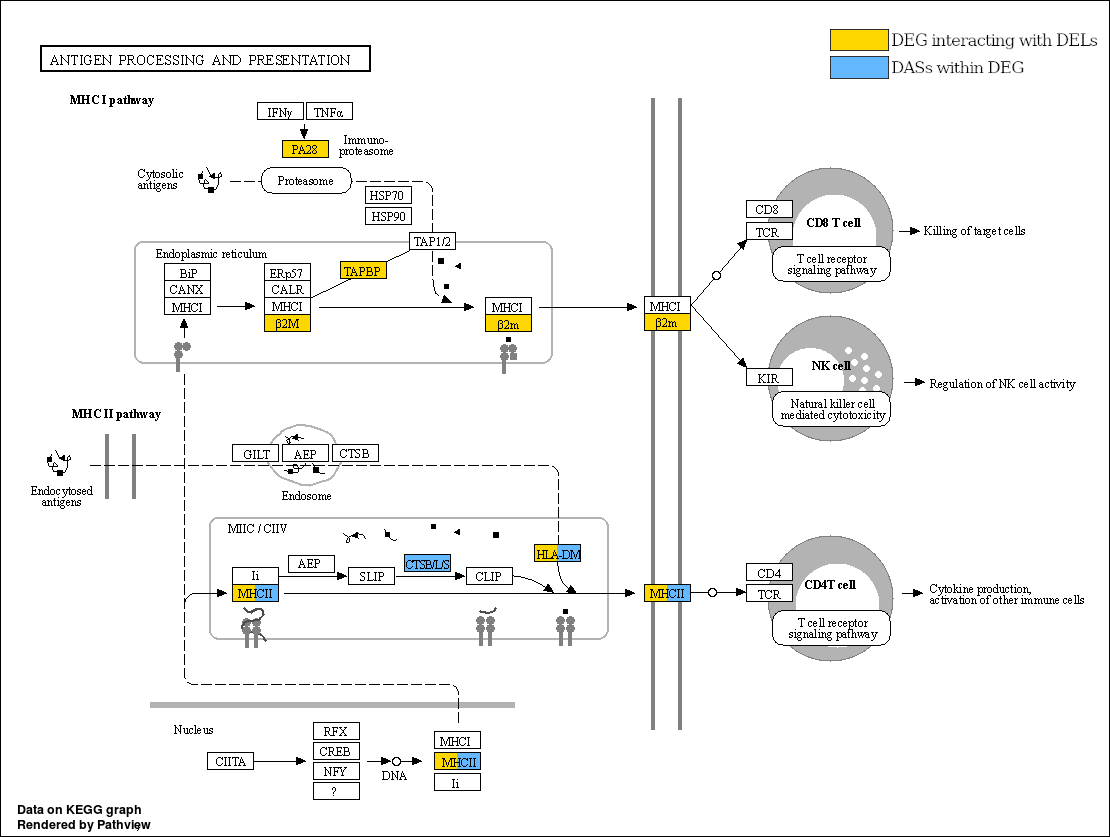

Supplement: Supplementary file 1 [file cells-11-00715-s001.zip › Supplementary_materials/Figure_S10.png]

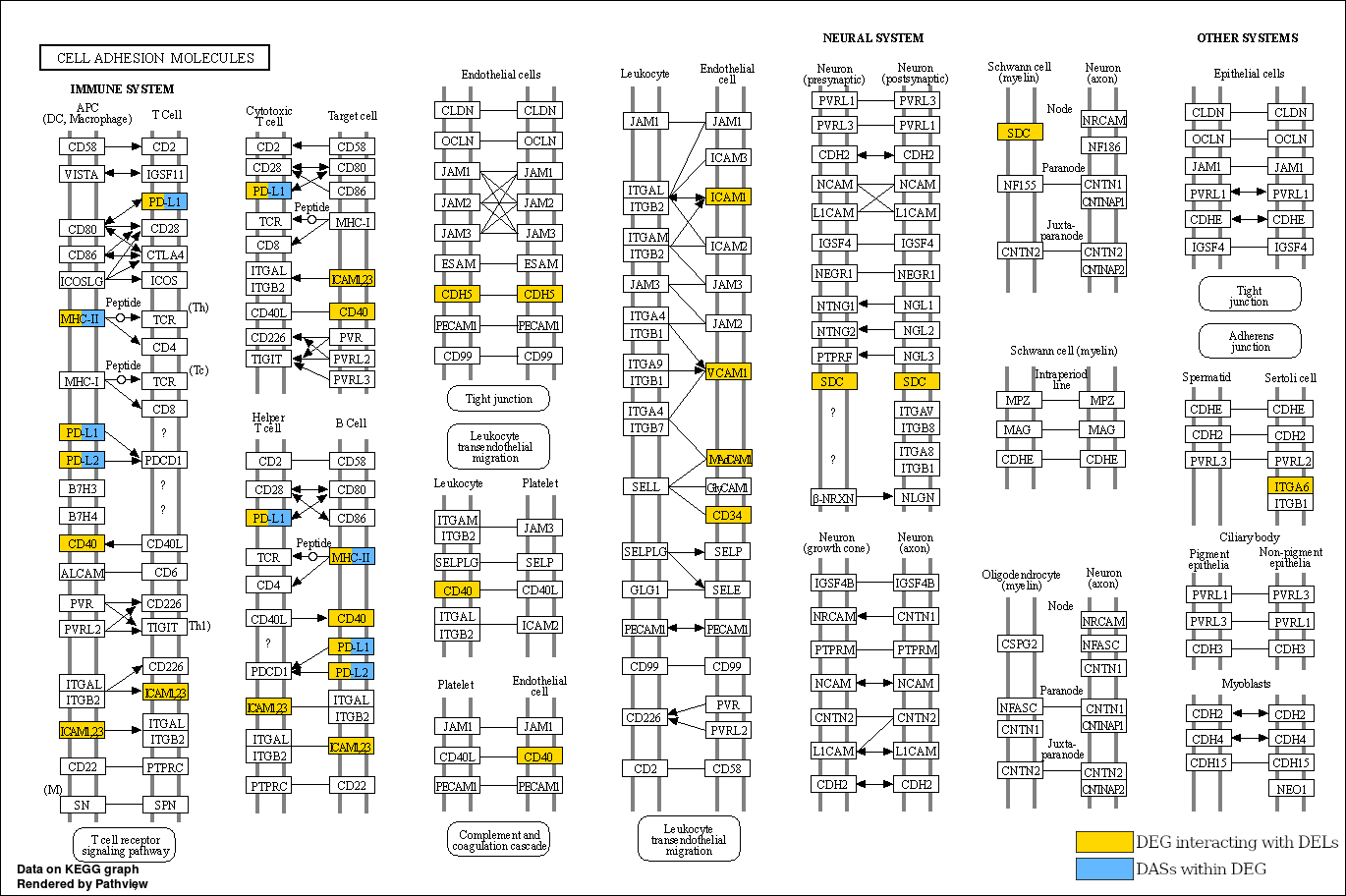

Supplement: Supplementary file 1 [file cells-11-00715-s001.zip › Supplementary_materials/Figure_S11.png]

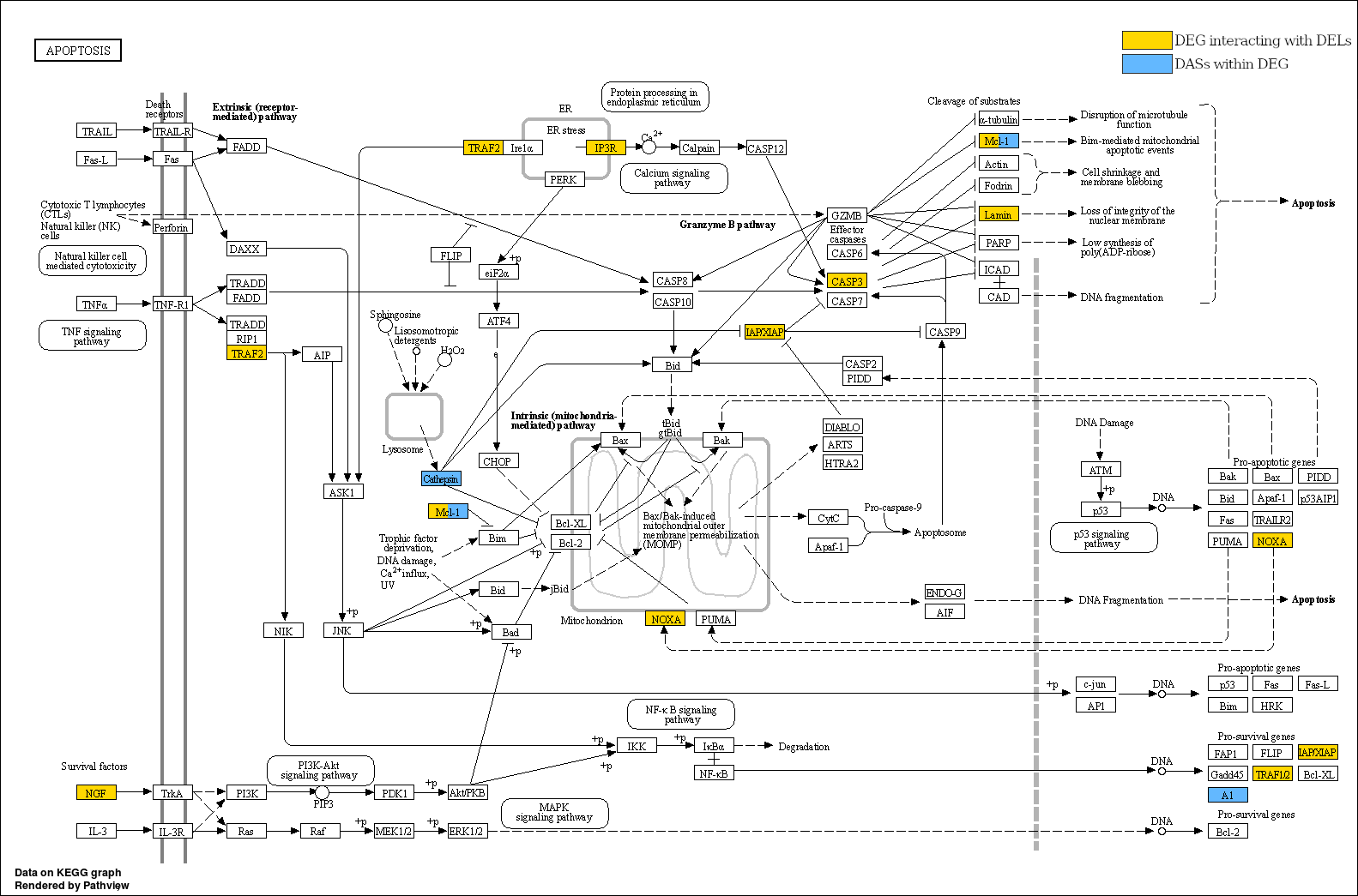

Supplement: Supplementary file 1 [file cells-11-00715-s001.zip › Supplementary_materials/Figure_S12.png]

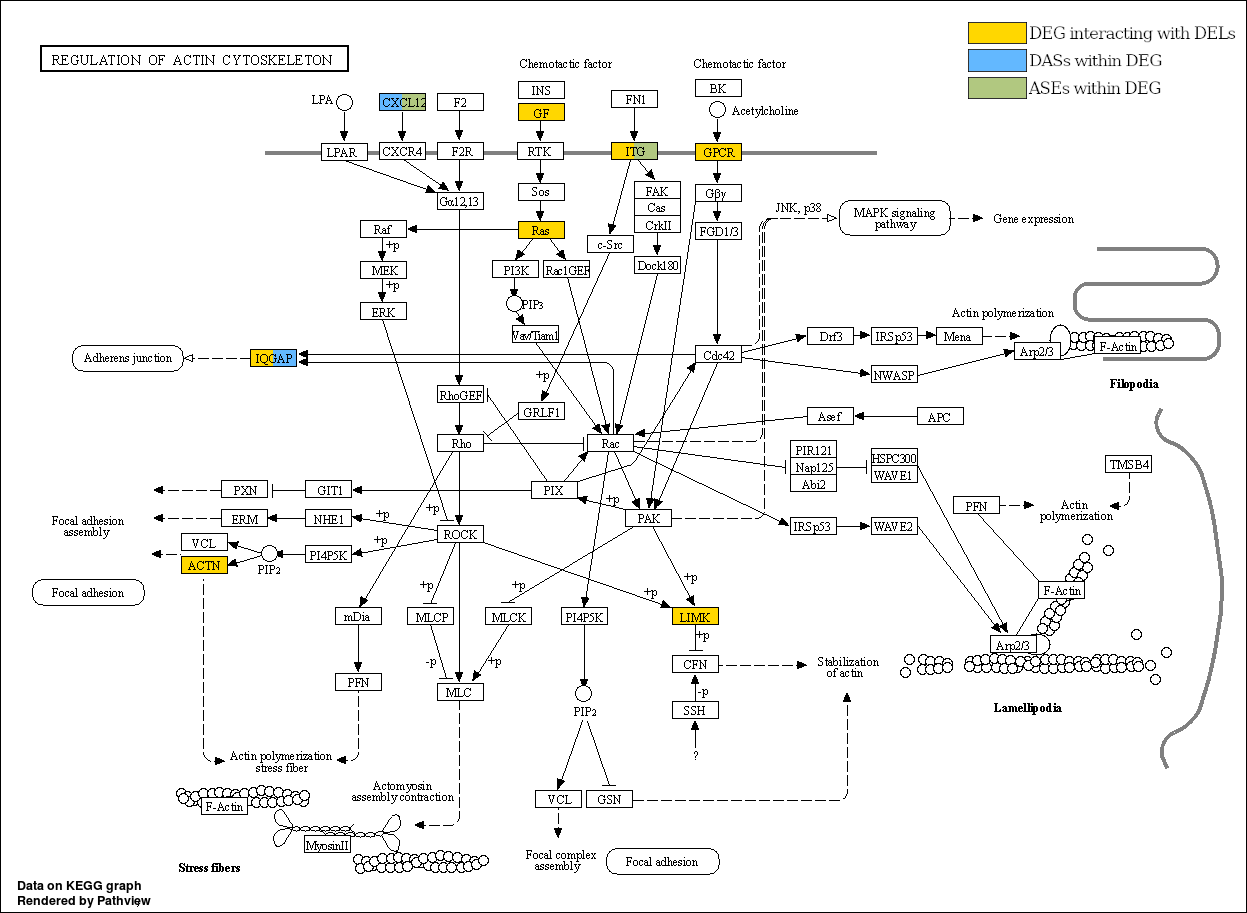

Supplement: Supplementary file 1 [file cells-11-00715-s001.zip › Supplementary_materials/Figure_S13.png]

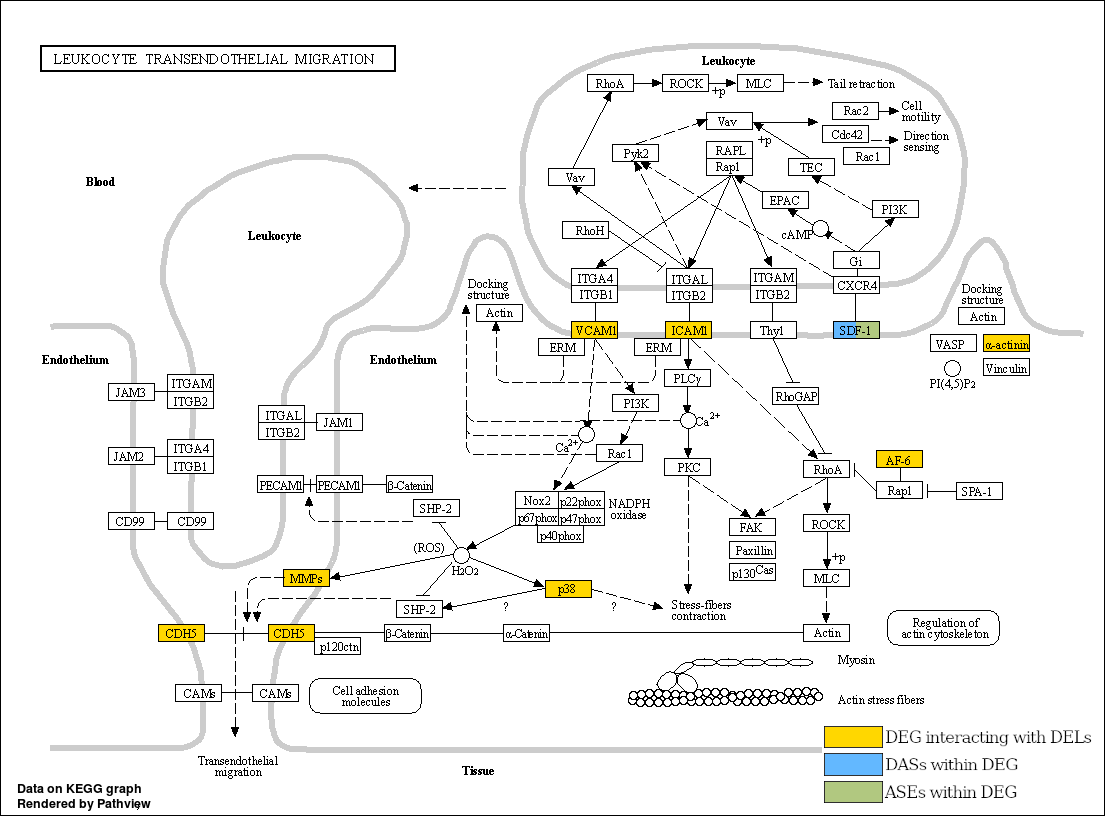

Supplement: Supplementary file 1 [file cells-11-00715-s001.zip › Supplementary_materials/Figure_S14.png]

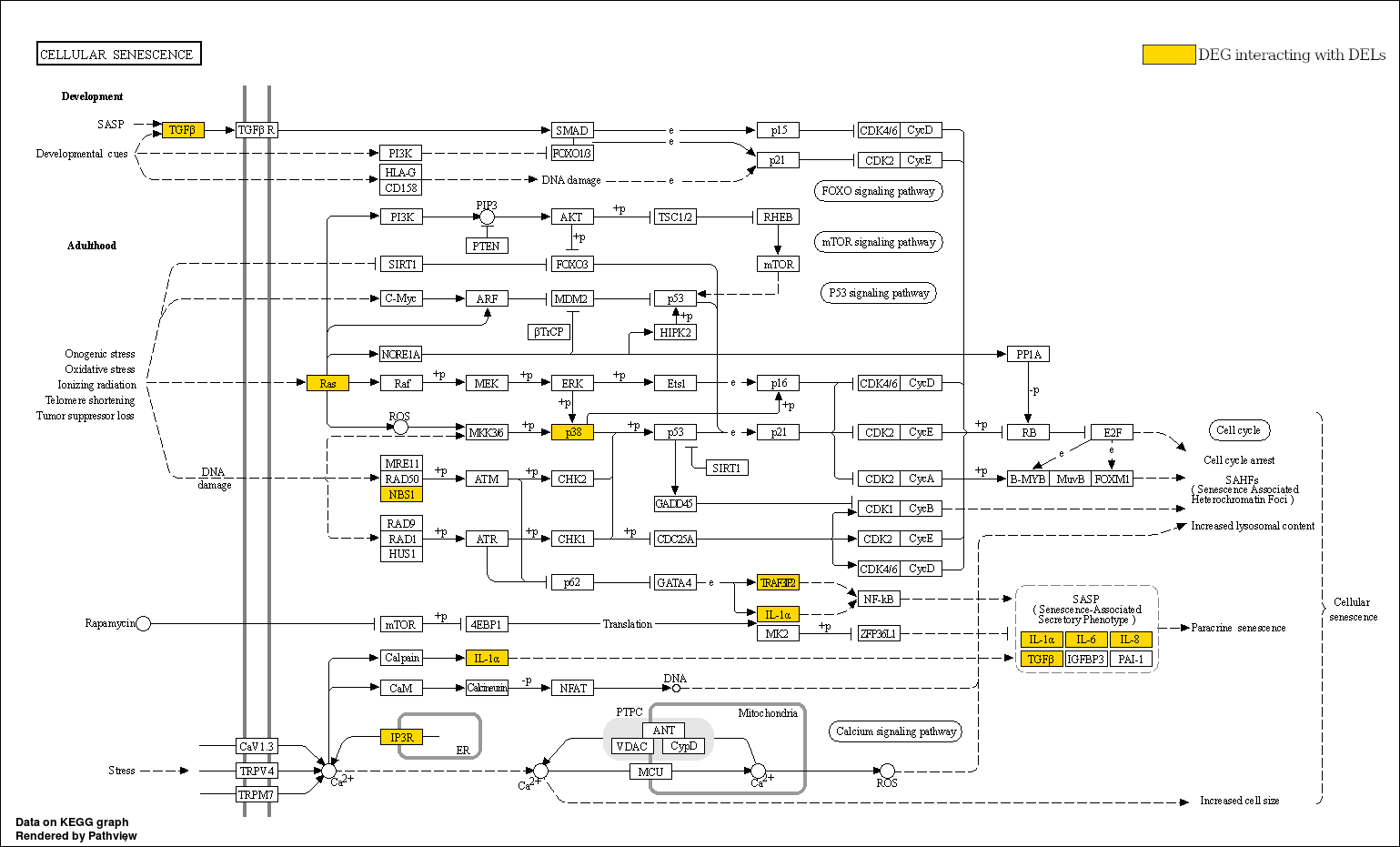

Supplement: Supplementary file 1 [file cells-11-00715-s001.zip › Supplementary_materials/Figure_S15.png]

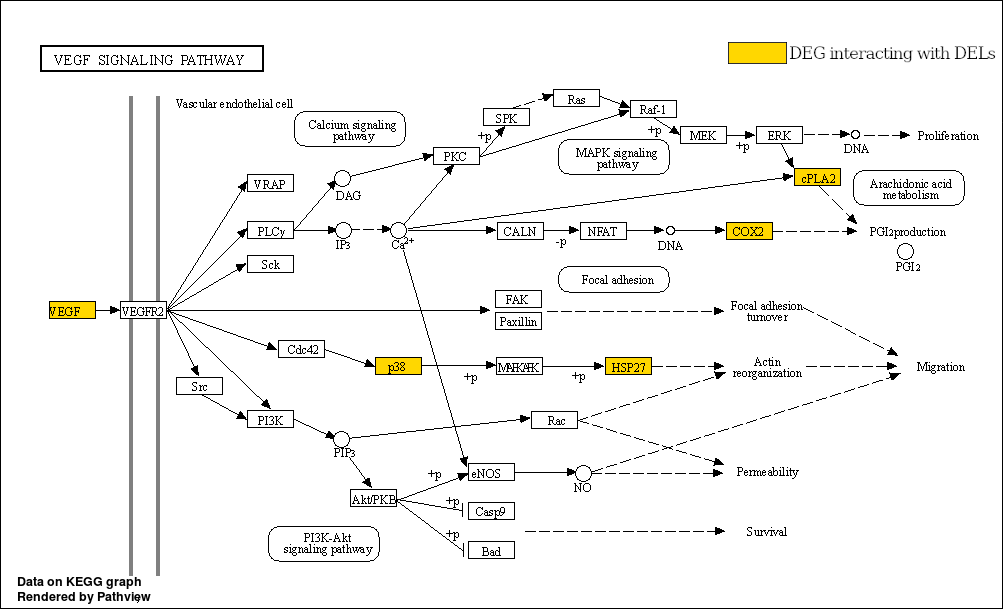

Supplement: Supplementary file 1 [file cells-11-00715-s001.zip › Supplementary_materials/Figure_S16.png]

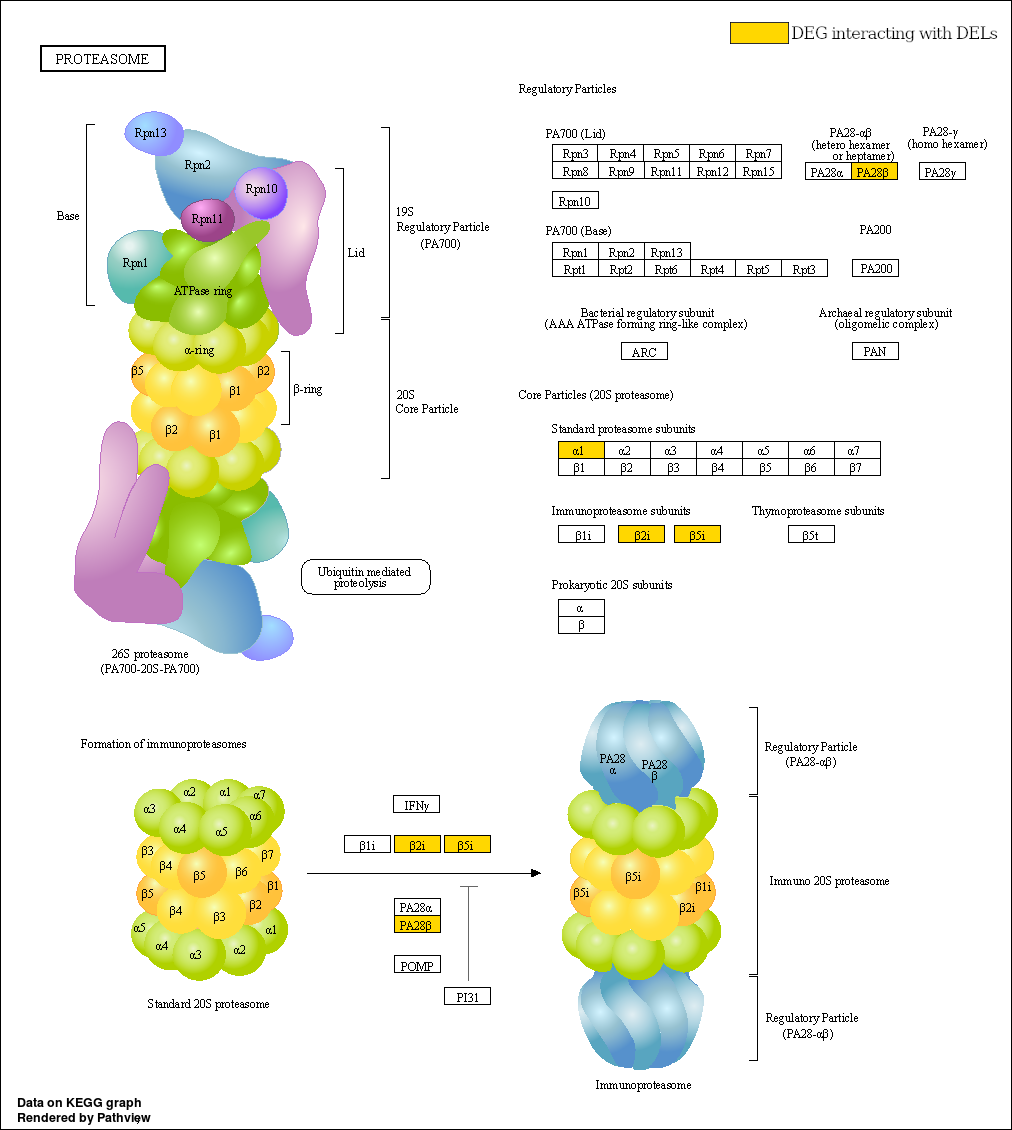

Supplement: Supplementary file 1 [file cells-11-00715-s001.zip › Supplementary_materials/Figure_S17.png]

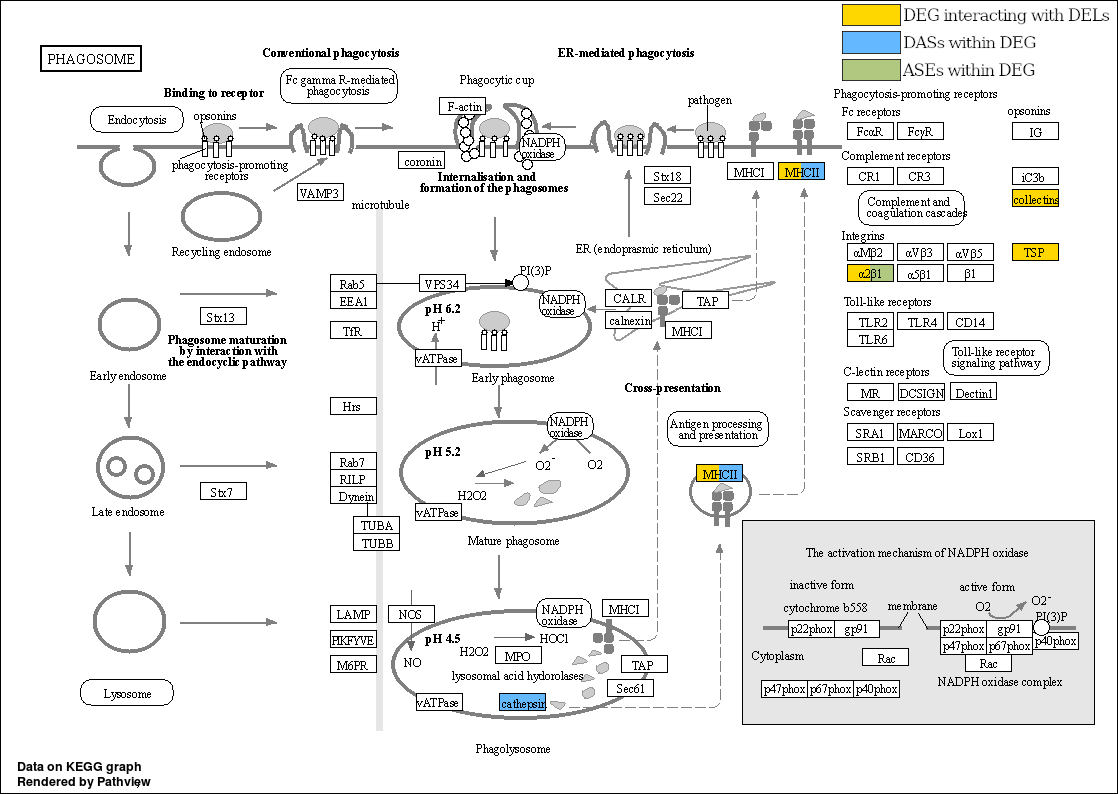

Supplement: Supplementary file 1 [file cells-11-00715-s001.zip › Supplementary_materials/Figure_S18.png]

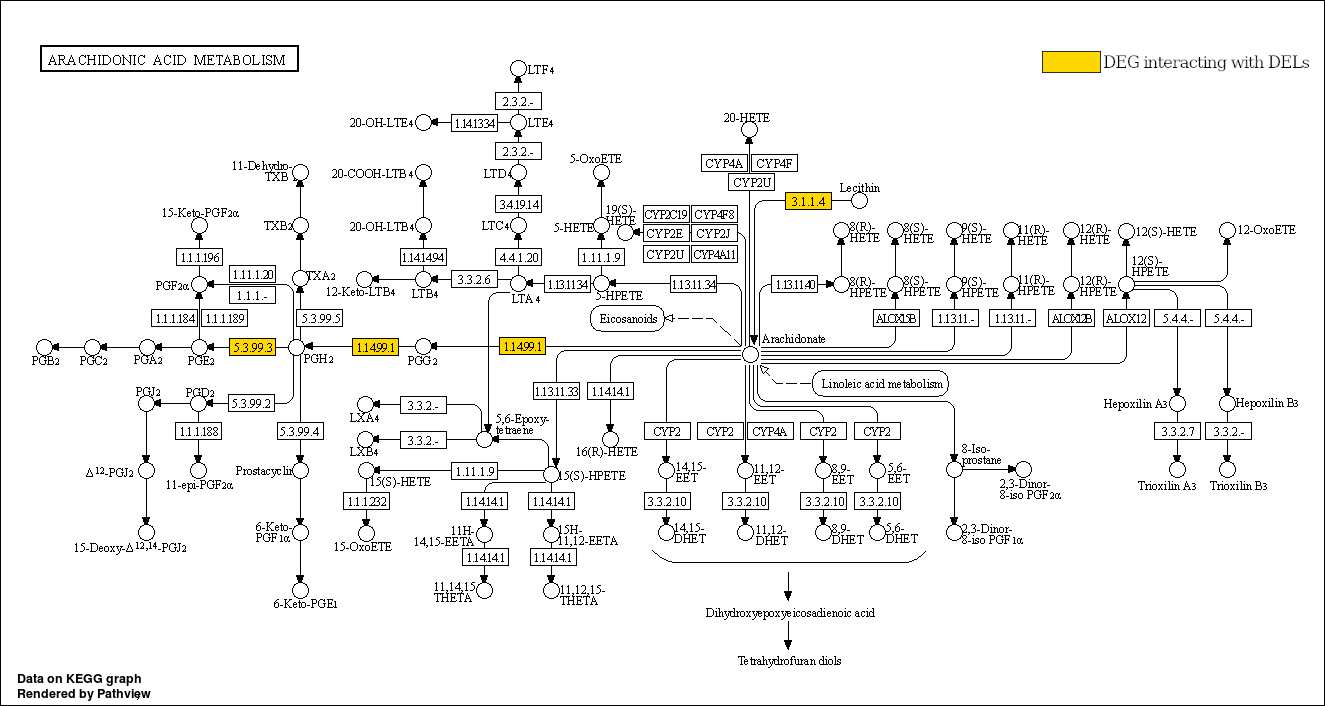

Supplement: Supplementary file 1 [file cells-11-00715-s001.zip › Supplementary_materials/Figure_S19.png]

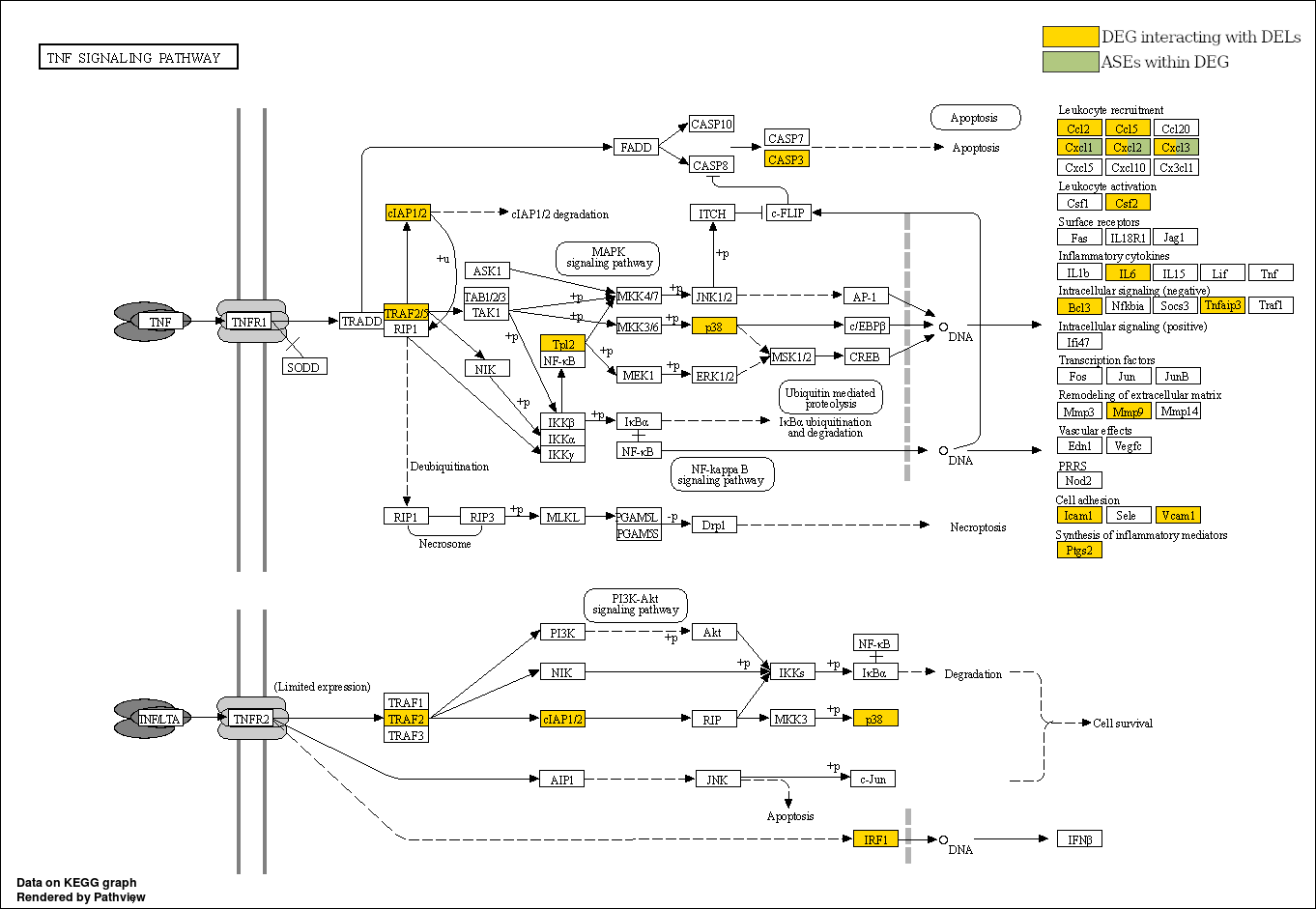

Supplement: Supplementary file 1 [file cells-11-00715-s001.zip › Supplementary_materials/Figure_S2.png]

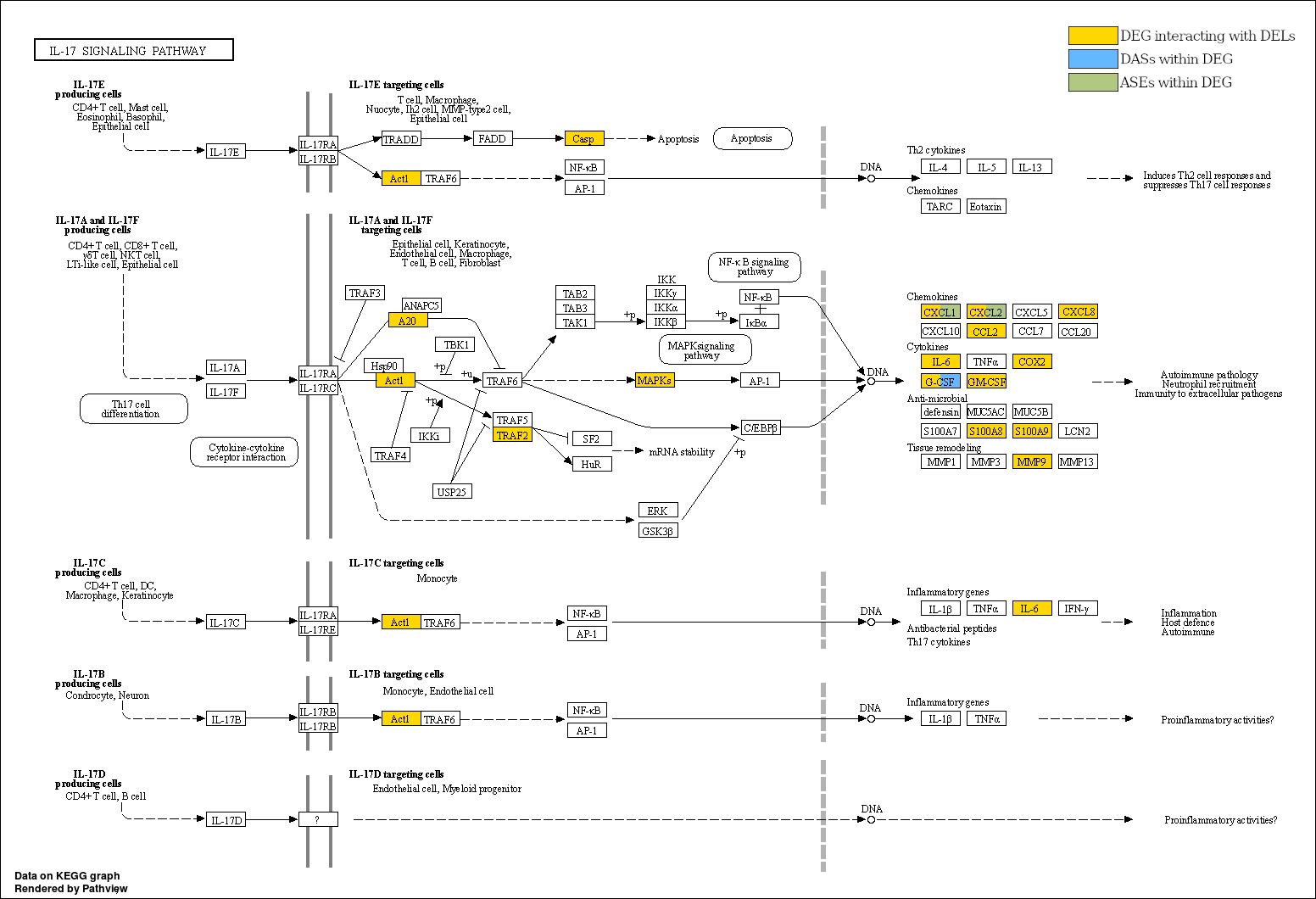

Supplement: Supplementary file 1 [file cells-11-00715-s001.zip › Supplementary_materials/Figure_S3.png]

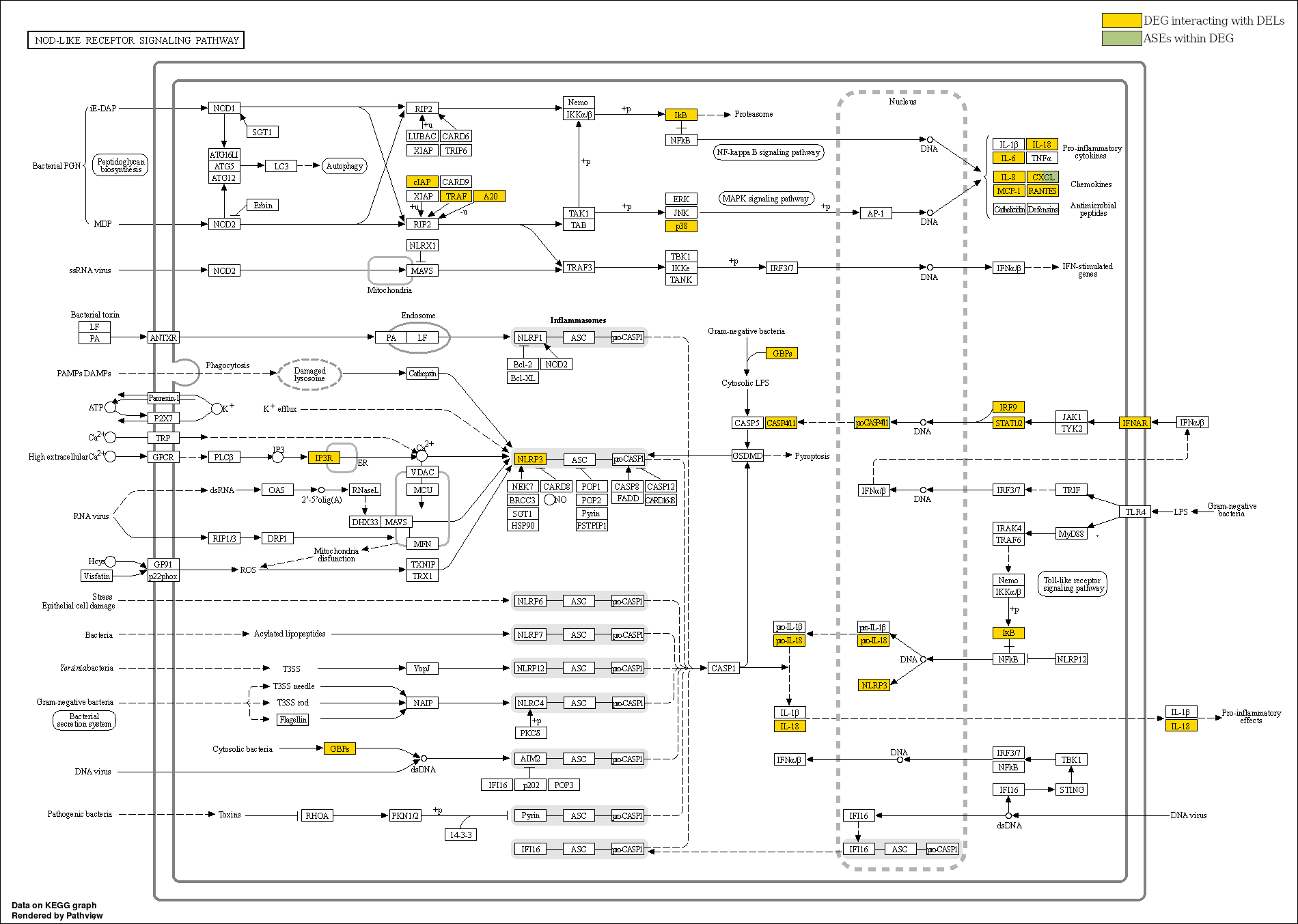

Supplement: Supplementary file 1 [file cells-11-00715-s001.zip › Supplementary_materials/Figure_S4.png]

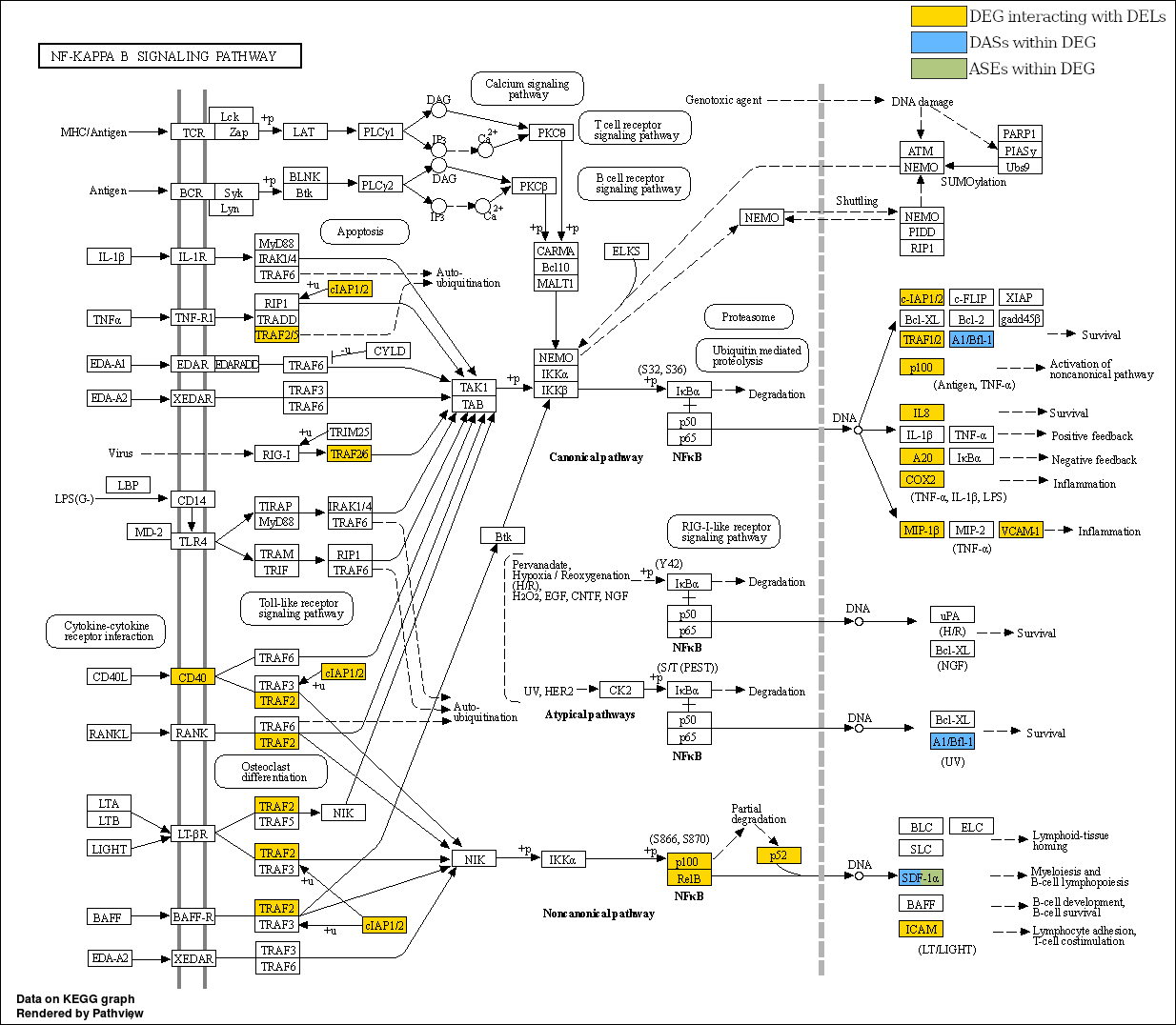

Supplement: Supplementary file 1 [file cells-11-00715-s001.zip › Supplementary_materials/Figure_S5.png]

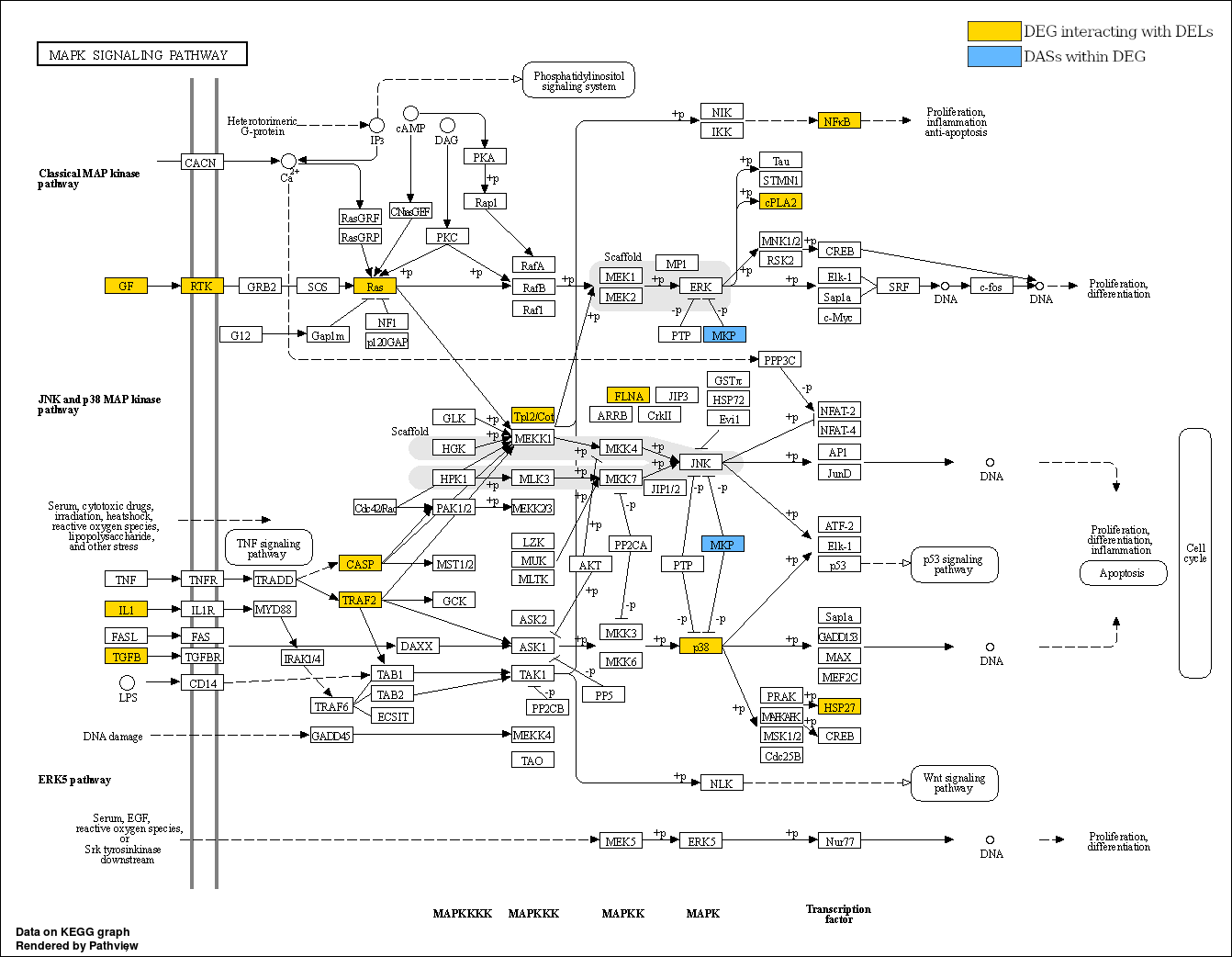

Supplement: Supplementary file 1 [file cells-11-00715-s001.zip › Supplementary_materials/Figure_S6.png]

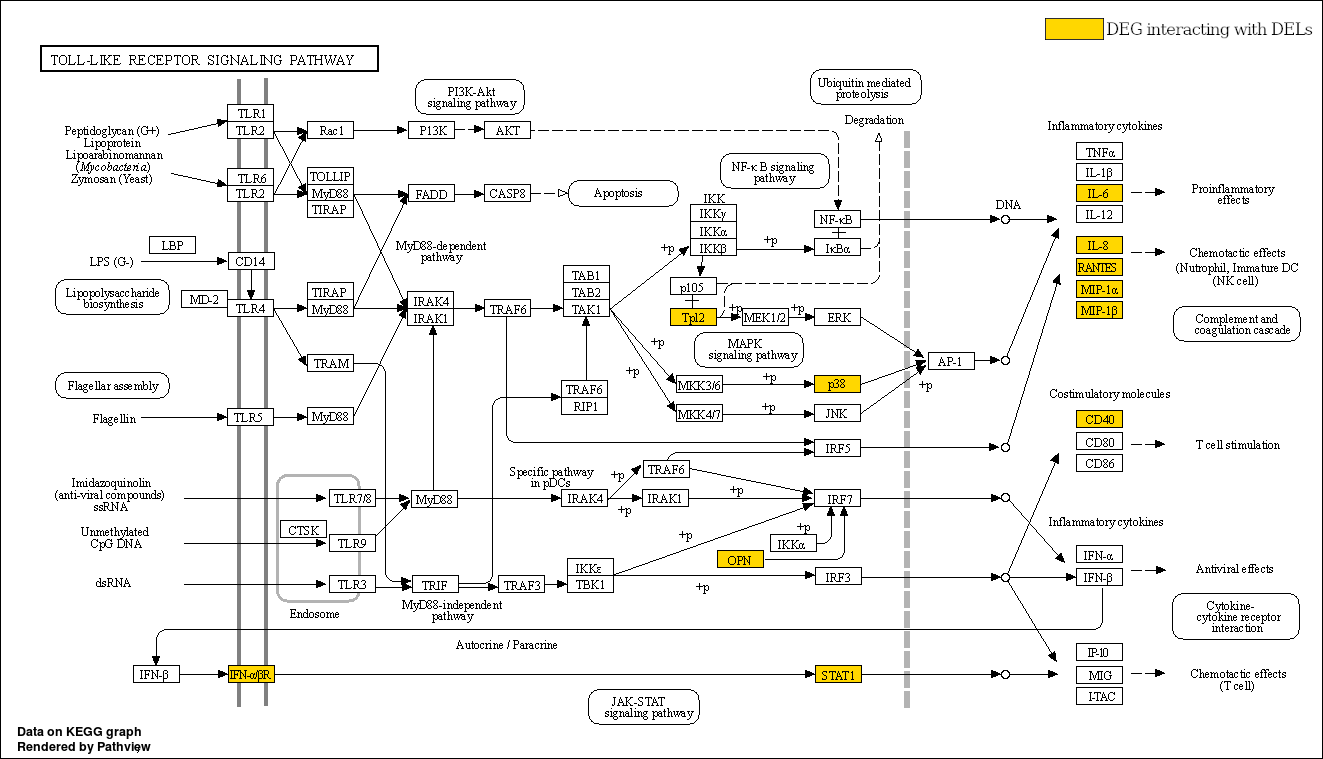

Supplement: Supplementary file 1 [file cells-11-00715-s001.zip › Supplementary_materials/Figure_S7.png]

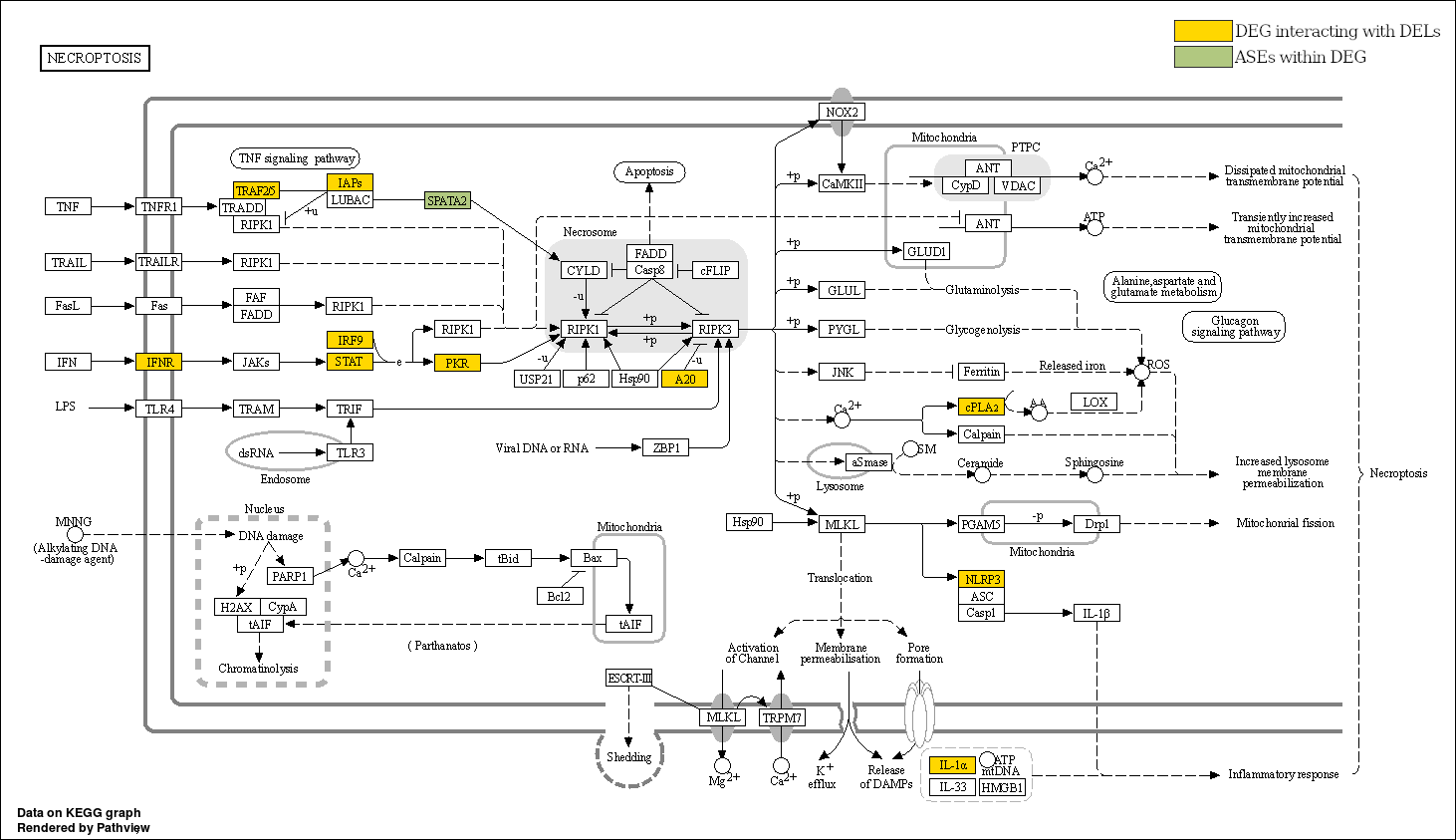

Supplement: Supplementary file 1 [file cells-11-00715-s001.zip › Supplementary_materials/Figure_S8.png]

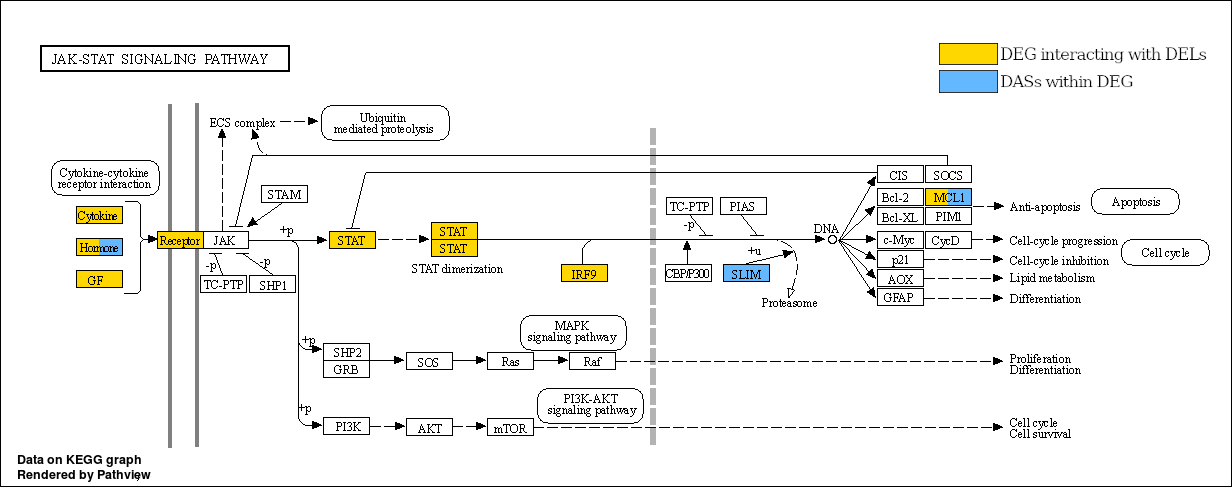

Supplement: Supplementary file 1 [file cells-11-00715-s001.zip › Supplementary_materials/Figure_S9.png]
